# Predictors of tuberculosis mortality among patients on treatment: A retrospective cohort study at a rural general hospital in Zambia

**DOI:** 10.1371/journal.pone.0345056

**Published:** 2026-06-24

**Authors:** Collins Mukubesa, Lukundo Siame, Malan Malumani, Faceroy Nkole, Propheria C. Lwiindi, Matenge Mutalange, Geofrey Mupeta, Memory Ngosa, Martin Chakulya, Benson M. Hamooya, Chitalu Chanda

**Affiliations:** 1 Maamba General Hospital, Sinazongwe, Zambia; 2 Department of Public Health, Mulungushi University School of Medicine and Health Sciences, Livingstone, Zambia; 3 Department of Internal Medicine, Livingstone University Teaching Hospital, Livingstone, Zambia; 4 Ministry of Health, Kazungula District Health Office, Kazungula, Zambia; 5 Rusangu University, Monze, Zambia; 6 Department of Internal Medicine, University Teaching Hospital, Lusaka, Zambia; University of Sydney, AUSTRALIA

## Abstract

**Background:**

Despite decades of global efforts and the availability of curative and preventive measures, tuberculosis (TB) continues to be the world’s leading infectious disease cause of mortality, particularly in resource-limited settings. However, in developing countries, there is limited evidence on determinants of mortality during TB treatment, making it difficult to design targeted interventions. This study aimed to determine the incidence and factors associated with mortality among people diagnosed with TB at a rural health facility in Zambia.

**Methods:**

We conducted a retrospective cohort study among 316 individuals who started TB treatment between May 2019 and March 2024. Participants were followed from treatment initiation until a treatment outcome was recorded, with a maximum follow-up time of 12 months. Demographic and clinical information were collected from medical records using a data collection tool via KoboTool software. The primary outcome was mortality. We used a multivariable Cox regression model to identify factors associated with TB mortality using Stata version 15.

**Results:**

The participants had a median age of 41 years (IQR: 32–54) and a median follow-up of 174 days (IQR: 155–191). Overall, 19.0% (n = 60; 95% CI: 14.8–23.8) of participants died, corresponding to an incidence rate of 42.1 per 100 person-years based on 51,968 person-days of follow-up (95% CI: 31.6–54.0). Age (adjusted hazard ratio (aHR): 1.017, 95% CI: 1.000–1.033, P = 0.042), clinically diagnosed TB had a significantly higher risk of mortality compared to those with bacteriologically confirmed TB (aHR: 1.97, 95% CI: 1.14–3.41, p = 0.015), and People living with HIV (aHR: 1.84, 95% CI: 1.03–3.29, P = 0.039) were significantly associated with increased mortality. While being on family-based directly observed therapy (DOT) (aHR: 0.42, 95% CI: 0.24–0.74, P = 0.003) and every unit increase in baseline weight (aHR: 0.96, 95% CI: 0.92–0.99, p = 0.004) was associated with a reduced risk of mortality.

**Conclusion:**

This study reveals a high incidence of TB-related mortality in a rural setting, which is significantly influenced by demographic and clinical determinants. The findings highlight the need for closer evaluation of patients with clinically diagnosed TB and strengthening family-based DOT as strategies to reduce mortality.

## Introduction

Tuberculosis (TB) remains one of the leading causes of mortality from infectious diseases worldwide, despite the availability of effective diagnostic tools and treatment [1]. In 2021, an estimated 10 million people developed TB, resulting in approximately 1.3 million deaths among HIV-negative individuals and 214,000 deaths among those co-infected with HIV [2]. The persistence of TB-related deaths highlights the ongoing challenges in TB control, particularly in low- and middle-income countries [3]. Zambia continues to experience a significant burden of TB and is classified among the 30 high TB-burden countries globally, ranking 21st in prevalence [4]. Although the national TB mortality rate has declined from 30 per 100,000 population in 2015–21 per 100,000 in 2021, TB remains a major public health problem [4].

Evidence suggests that TB mortality is associated with a variety of factors, with HIV co-infection being the most common, while advanced age, male sex, extrapulmonary TB, and delayed diagnosis are also important contributing factors in Zambia [5,6]. Recent reports suggest that deaths among TB patients in Sub-Saharan Africa occur within the first two months of treatment initiation, reflecting a critical window of vulnerability when patients are most unstable [5].

Persistent TB mortality in high-burden settings such as Zambia may be attributed to multiple patient-level and health system factors that vary by region [5]. Rural and semi-urban hospitals often face unique challenges, including limited diagnostic capacity, inadequate human resources, and poor treatment adherence monitoring, which can contribute to adverse outcomes [5]. Moreover, co-infection with HIV continues to exacerbate TB mortality, as immune suppression increases susceptibility to severe disease and treatment failure [7]. Emerging evidence also highlights the growing contribution of non-communicable diseases (NCDs) such as diabetes mellitus, chronic obstructive pulmonary disease (COPD) and undernutrition to TB mortality as they all impair immune function and complicate treatment outcomes [5]. The intersection of TB with NCDs represents a new frontier in TB control, particularly in low-resource settings where integrated management is still limited [8]. Health system barriers such as delayed initiation of Antiretroviral therapy (ART) in co-infected patients, inconsistent implementation of Directly Observed Therapy (DOT) and lack of nutritional support have been linked to poor outcomes [5]. Understanding these local determinants and strengthening these aspects of TB care is crucial, as it could significantly reduce preventable deaths. National statistics often mask the contextual differences that influence TB outcomes. Therefore, site-specific studies are essential to identify local predictors of mortality, evaluate existing care models and inform evidence-based strategies and interventions to strengthen local TB control efforts and enhance patient survival.

In line with the World Health Organization’s End TB Strategy, which aims to reduce TB-related deaths by 95% by 2035 [9], this study sought to determine the predictors of mortality and associated factors among adult patients diagnosed with TB and receiving treatment at Maamba General Hospital in southern Zambia.

## Methodology

### Study design and site

This was a retrospective cohort study among adolescents and adults aged 15 years patients diagnosed with tuberculosis at the Chest Clinic of Maamba General Hospital between May 2019 and March 2024. The clinic manages patients of all ages diagnosed with TB. The presumptive TB cases are recorded in a presumptive register. Those diagnosed with TB are then entered in the TB treatment register and treated according to recommended guidelines [10]. Treatment outcomes are recorded by healthcare workers in the TB treatment register and patient cards. The outcomes are categorized according to national TB guidelines as ‘cured’, ‘treatment completed’, ‘died’, ‘treatment failed’, or ‘lost to follow-up’ (LTFU).

### Study population and sampling methods

The study population consisted of adolescents and adults diagnosed with drug-sensitive tuberculosis (TB) who initiated TB treatment at the clinic between May 2019 and March 2024. A total of 483 medical records were reviewed. A census sampling approach was used, whereby all patient records listed in the tuberculosis treatment registers during the study period (May 2019 to March 2024) were reviewed and screened for eligibility.

### Inclusion and exclusion criteria

**Fig 1:**
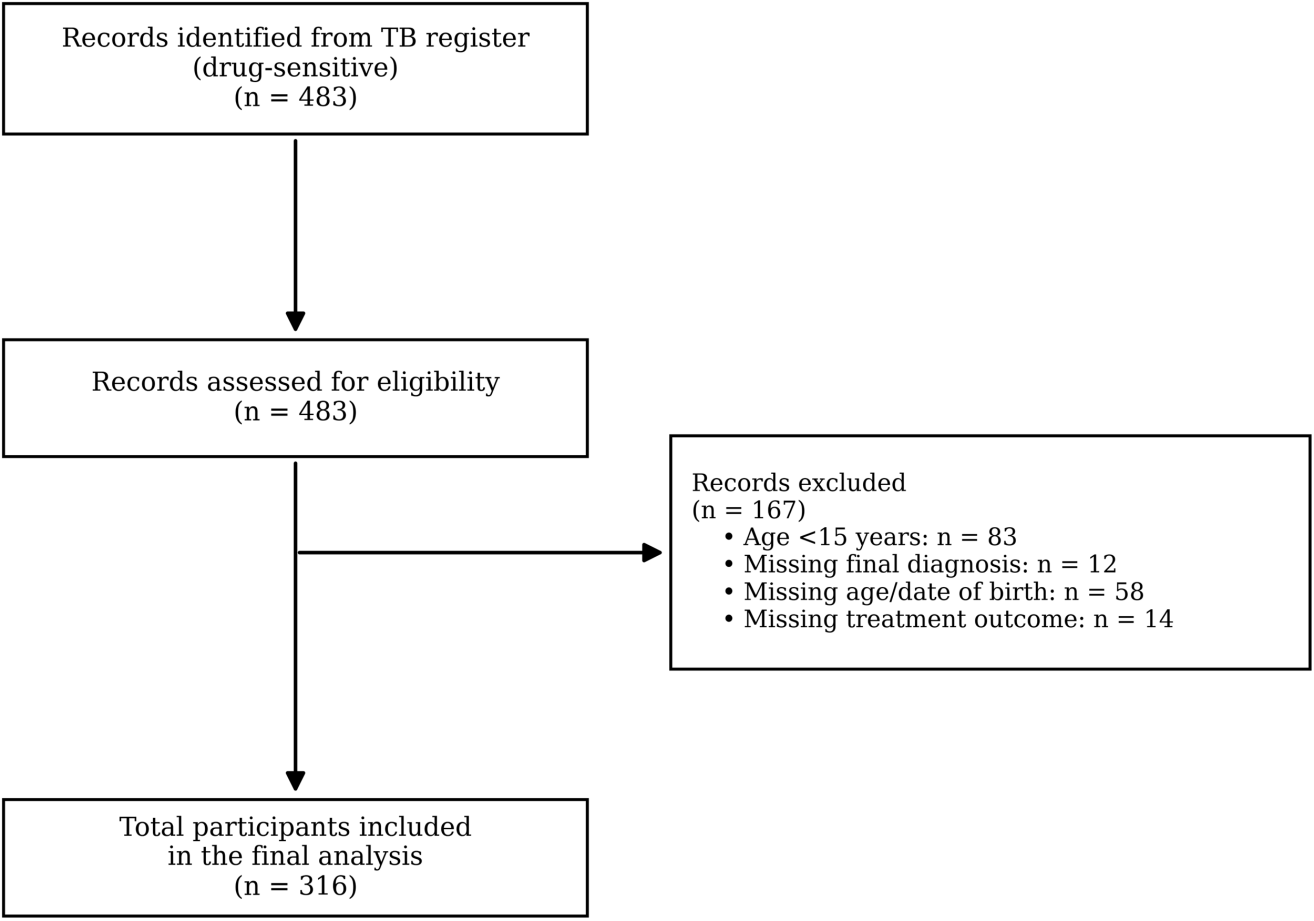
Flow diagram showing the selection of study participants from the TB treatment register at Maamba General Hospital, May 2019 – March 2024.

Eligible participants were adolescents and adults aged ≥15 years who were diagnosed with drug-sensitive tuberculosis, initiated TB treatment at the clinic between May 2019 and March 2024 and had a documented tuberculosis treatment outcome. Children aged <15 years (n = 83) were excluded at the eligibility assessment stage in line with national and international tuberculosis guidelines, which manage pediatric TB separately from adolescent and adult TB [11]. Additional exclusions included records without a confirmed final tuberculosis diagnosis (n = 12), records with missing or undocumented dates of birth that precluded age calculation (n = 58), and records missing final tuberculosis treatment outcomes (n = 14). After applying these criteria, 316 records were included in the final analytic cohort (see Fig 1).

### Variables in the study

The outcome variable was binary all-cause mortality during tuberculosis treatment, defined as any death recorded in the TB treatment register after treatment initiation and before treatment completion or censoring. Participants were classified as “died” if death occurred during the treatment period. All other treatment outcomes, including cured, treatment completed, treatment failure, lost to follow-up, or transfer-out, were classified as “did not die.” Deaths were recorded as part of routine programmatic follow-up by clinicians, and cause-specific attribution of death was not performed.

Predictor variables included demographic parameters (age, sex, residence, and occupation), clinical parameters (HIV status, type of tuberculosis, treatment regimen, co-trimoxazole therapy, and patient category), and baseline laboratory parameters (platelet count, hemoglobin, white blood cell count, CD4 count, alanine aminotransferase [ALT], and aspartate aminotransferase [AST]). Dates of tuberculosis treatment initiation and treatment outcome were also recorded.

Baseline laboratory values were defined as tests performed within 14 days before or after tuberculosis treatment initiation; when multiple measurements were available, the result closest to the treatment start date was used.

### Operational definitions

1. Clinically diagnosed tuberculosis was defined in accordance with the Zambia National Tuberculosis and Leprosy Programme guidelines as a case that does not fulfil the criteria for bacteriological confirmation but is diagnosed with tuberculosis by a qualified clinician and initiated on a full course of anti-tuberculosis treatment. This includes cases diagnosed based on compatible clinical features and/or chest radiographic abnormalities suggestive of tuberculosis, as well as extrapulmonary tuberculosis cases without laboratory confirmation [10].
2. Bacteriologically confirmed tuberculosis was defined as a case with a biological specimen positive for *Mycobacterium tuberculosis* by smear microscopy, culture, or WHO-approved rapid diagnostic tests, including Xpert MTB/RIF, line probe assay, or lateral flow urine lipoarabinomannan (LF-LAM) assay [10].
3. Directly observed therapy (DOT) was implemented in accordance with national tuberculosis treatment policy. Facility-based DOT referred to treatment intake observed by a healthcare worker at a health facility. Family-based DOT referred to treatment intake observed by a designated household member or treatment supporter at home, as documented in the TB treatment register [10].
4. Treatment outcomes were defined according to national guidelines. Successful outcomes included patients who were cured or completed treatment [10]. Cure was defined as bacteriologically confirmed pulmonary TB with documented response to treatment and no evidence of failure, while treatment completion referred to patients who completed therapy without meeting criteria for cure or failure [10]. Unsuccessful outcomes included treatment failure, lost to follow-up, or death [10]. Death was defined as death from any cause before or during treatment, and lost to follow-up as treatment interruption for two consecutive months or more [10].
5. Tuberculosis treatment guideline followed the Zambia National Tuberculosis and Leprosy Programme guidelines, with patients receiving either a standard 6-month regimen for non-severe TB or an extended 12-month regimen for severe forms, including tuberculous meningitis and osteoarticular/spinal TB. Drug dosing was based on national weight-band guidelines [10].

### Data collection procedure

Demographic, clinical, and laboratory data were collected by trained research assistants using a structured electronic data capture tool developed in the Kobo Toolbox application. Information was retrospectively abstracted from the paper-based drug-susceptible tuberculosis treatment register for patients who initiated treatment between May 2019 and March 2024. Data collection was conducted during the second and third weeks of September 2024. The completeness and accuracy of the records were reviewed at the end of each data collection session to ensure data integrity. As part of quality control, 10% of patient records were re-abstracted using systematic random sampling and compared with the original entries; any discrepancies identified were discussed and resolved.

### Data analysis

Data were analysed using Stata version 15. Categorical variables were summarised using frequencies and proportions, and continuous variables using medians and interquartile ranges. Time to mortality was defined as the interval from tuberculosis treatment initiation to death or censoring. Survival probabilities were estimated using Kaplan–Meier methods, and Cox proportional hazards regression was used to identify predictors of mortality, with unadjusted and adjusted hazard ratios and 95% confidence intervals reported.

Time-to-death was defined as the number of days from tuberculosis treatment initiation to death or censoring. Participants were censored at treatment completion, loss to follow-up, transfer-out, or administrative censoring at 12 months. Transfers out were censored at the date of last documented clinic visit. Person-time at risk was calculated as the interval between treatment initiation and death or censoring for each participant and summed across individuals. Total person-time was expressed in person-days and converted to person-years by dividing by 365.25.

Missing data were assessed for all variables (Supplementary Table S1 in S1 File). Missingness among covariates included in the regression models was low (<6%), with no missing outcome data; therefore, complete-case analysis was used. Variables with substantial missingness (including selected laboratory measures and CD4 count) were excluded from multivariable modelling, and multiple imputations were not performed.

Variables were selected for inclusion in the multivariable model based on prior evidence of association with tuberculosis mortality, statistical significance in the bivariate analysis (p < 0.20), and clinical relevance [6]. An enter method was used, whereby all selected variables were entered simultaneously into the multivariable Cox regression model. The proportional hazards assumption was assessed using Schoenfeld residuals (χ² = 4.58, df = 8, p = 0.80), and no individual covariate demonstrated non-proportional hazards (all p > 0.05) (see supplementary table 2 in S2 File). Collinearity among exploratory variables was assessed using variance inflation factors (VIFs). All variables had VIF values close to 1 (mean VIF = 1.04).

The final multivariable Cox regression model included 284 participants with 54 mortality events, corresponding to approximately 7 events per variable, which is considered acceptable in observational survival analyses when covariates are selected a priori based on strong clinical justification [12]. Statistical significance was set at p < 0.05.

### Ethics

Ethical approval to conduct this study was obtained from the Mulungushi University Research Ethics (date of clearance 24th June 2024, ethics number: SMHS-MU2-2024-301). Given the retrospective nature of the study and the use of routinely collected programmatic records, the requirement for individual informed consent was waived by the ethics committee. All data analyzed were de-identified to ensure confidentiality, and no patient was identifiable during or after data collection.

## Results

### Demographics and clinical characteristics

The median age of the participants was 41 years (interquartile range [IQR]: 32–54; n = 316), with the majority being male (n = 201/316; 63.6%) and informally employed (n = 299/316; 94.6%). Pulmonary TB was the most common type, affecting 91.1% of participants (n = 288). Most cases were newly diagnosed (n = 262, 83.2%), and the majority had bacteriologically confirmed TB (n = 205, 65.9%). A total of 62.5% (n = 195) had Gene Xpert testing, 10.4% (n = 32) had smear, and 17.5% (n = 55) had urine lam test done. The median weight at the start of treatment was 52 kg (IQR: 46–58), which increased to 57.5 kg (IQR: 50–66) at the end of treatment. The majority of participants (n = 195, 61.9%) had their treatment observed by family members. The median hemoglobin level was 11.1 g/dL (IQR: 9.1–13), while the median white blood cell (WBC) count was 5.6 × 10⁹/L (IQR: 3.9–8.6). The median platelet count was 270 × 10⁹/L (IQR: 185–385). The median creatinine level was 91.9 μmol/L (IQR: 81.4–122.5), and the median urea level was 4.1 mmol/L (IQR: 3.1–6.3). The median CD4 count was 331 cells/mm³ (IQR: 162–523). Additionally, the median ALT level was 22.4 IU/L (IQR: 15.9–29.3), and the median AST level was 35 IU/L (IQR: 28.6–46.2), (Table 1).

**Table 1:** Baseline demographics, laboratory and clinical characteristics.

Variable	Available n	Missing n (%)	Median (IQR) or frequency (%)	Death: Yes (n = 60)	Death: No (n = 256)	p-value
**Age, years**	316	0 (0.0)	41 (32–54)	45 (36–57)	40.5 (30–52)	**0.022**
**Sex**	316	0 (0.0)				0.253
Male			201 (63.6)	42 (20.9)	159 (79.1)	
Female			115 (36.4)	18 (15.6)	97 (84.4)	
**Employment status**	316	0 (0.0)				0.624
Formal			17 (5.4)	4 (23.5)	13 (76.5)	
Informal			299 (94.6)	56 (18.7)	243 (81.3)	
**HIV status**	300	16 (5.1)				0.148
HIV-negative			200 (66.7)	34 (17.0)	166 (83.0)	
PLHIV			100 (33.3)	24 (24.0)	76 (76.0)	
**Type of tuberculosis**	316	0 (0.0)				0.176
Pulmonary			288 (91.1)	52 (18.1)	236 (81.9)	
Disseminated			28 (8.9)	8 (28.6)	20 (71.4)	
**Patient category**	316	0 (0.0)				0.350
New			262 (83.2)	48 (18.3)	214 (81.7)	
Relapse			41 (12.7)	10 (25.0)	30 (75.0)	
Transfer-in			13 (4.1)	1 (7.7)	12 (92.3)	
**Diagnosis type**	311	5 (1.6)				**0.011**
Bacteriological			205 (65.9)	30 (14.6)	175 (85.4)	
Clinical			106 (34.1)	28 (26.4)	78 (73.6)	
**DOT plan**	315	1 (0.3)				**<0.001**
Facility-based			120 (38.1)	37 (30.8)	83 (69.2)	
Family-based			195 (61.9)	23 (11.8)	172 (88.2)	
**Weight at treatment start, kg**	306	10 (3.2)	52 (46–58)	48 (43–53)	53 (47–60)	**<0.001**
**Weight at treatment end, kg**	108	208 (65.8)	57.5 (50–66)	58 (58–58)	57 (50–66)	0.941
**Hemoglobin, g/dL**	112	204 (64.6)	11.1 (9.1–13.0)	10.5 (8.7–13.0)	11.1 (9.1–13.0)	0.969
**WBC, × 10⁹/L**	111	205 (64.9)	5.6 (3.9–8.6)	5.4 (4.1–9.2)	5.6 (3.9–8.5)	0.919
**Platelets, × 10⁹/L**	108	208 (65.8)	270 (185–385)	317 (153–402)	267 (186–382)	0.779
**Creatinine, μmol/L**	70	246 (77.8)	91.6 (81.4–119.4)	90.1 (82.5–113.1)	92.0 (81.3–119.4)	0.789
**Urea, mmol/L**	53	263 (83.2)	4.1 (3.1–6.3)	3.9 (3.1–5.9)	4.1 (3.1–6.5)	0.474
**CD4 count†, cells/mm³**	58	242 (80.7)	331 (162–523)	256 (76–532)	346.5 (192.5–497)	0.885
**ALT, IU/L**	40	276 (87.3)	22.4 (15.9–29.3)	28.9 (27.8–30.0)	37.6 (28.7–46.3)	0.143
**AST, IU/L**	48	268 (84.8)	35.0 (28.6–46.2)	19.0 (16.0–20.5)	22.6 (15.7–30.2)	0.553

Footnotes: Data are presented as median (interquartile range) for continuous variables and frequency (%) for categorical variables.

Percentages are calculated using available observations per variable.

† CD4 count availability and missingness were calculated among participants living with HIV (n = 300).

Laboratory investigations were performed based on clinical indication and resource availability; missingness reflects routine programmatic practice rather than data recording errors.

Abbreviations: DOT = Directly Observed Therapy; PLHIV = People Living with HIV; WBC = White Blood Cell count; ALT = Alanine Aminotransferase; AST = Aspartate Aminotransferase.

### Relationship between mortality and other study variables

The cumulative mortality was 19.0% (n = 60; 95% CI: 14.8%–23.8%), corresponding to an incidence rate of 42.1 deaths per 100 person-years, based on 51,968 person-days of follow-up among 316 participants (95% CI: 31.6–54.0).Participants diagnosed with confirmed bacteriological TB had a lower mortality rate compared to those diagnosed clinically (14.6% vs. 26.4%, p = 0.011). Individuals who died were significantly older than those who survived, 45 vs. 40.5 years, p = 0.022. The median weight at the start of treatment was significantly lower among those who died compared to survivors (48 kg vs. 53 kg, p < 0.001). Mortality was higher among participants observed daily at the hospital facility compared to those observed daily by family (30.8% vs. 11.8%) (Table 1).

### Survival analysis of TB patients

**Fig 2:**
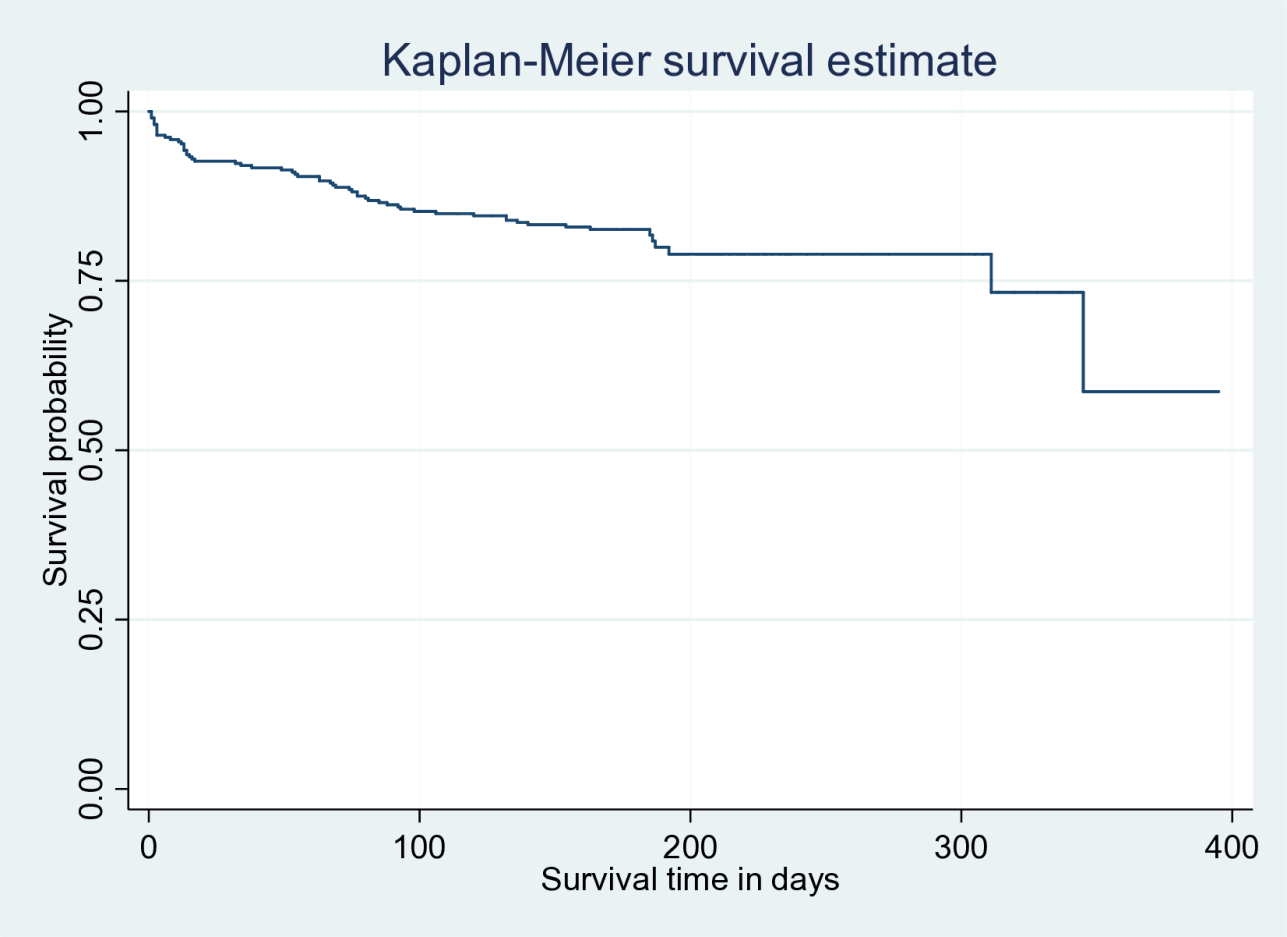
Kaplan-Meier Survival Curve Showing Overall Survival Among Tuberculosis Patients on Treatment at Maamba General Hospital (n = 316).

**Fig 3:**
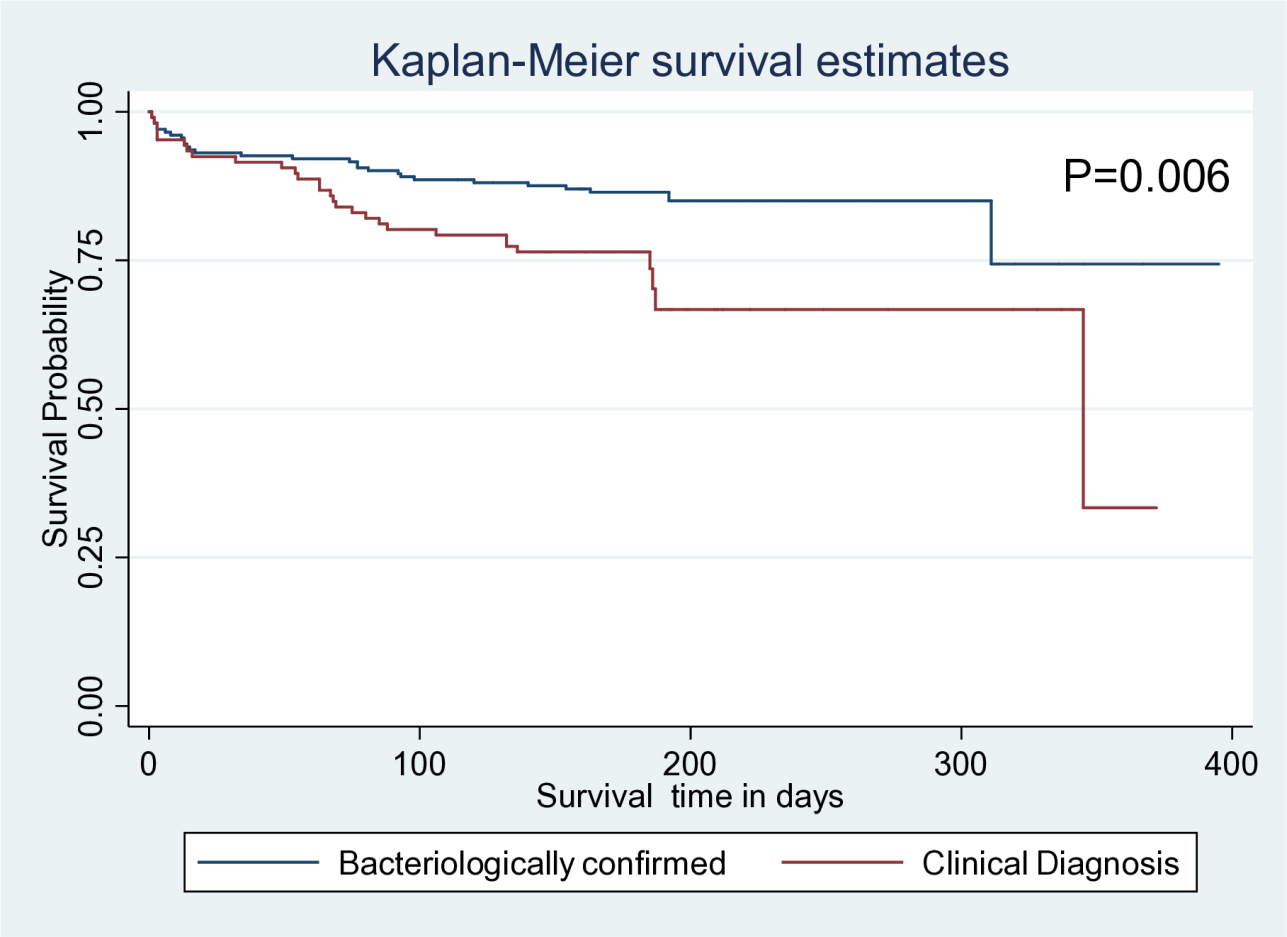
Kaplan–Meier survival curves showing cumulative survival probability among tuberculosis patients with bacteriologically confirmed TB (n = 205) versus clinically diagnosed TB (n = 106). Numbers at risk at selected time points are shown below the x-axis. Log-rank test p-value = 0.006.

**Fig 4:**
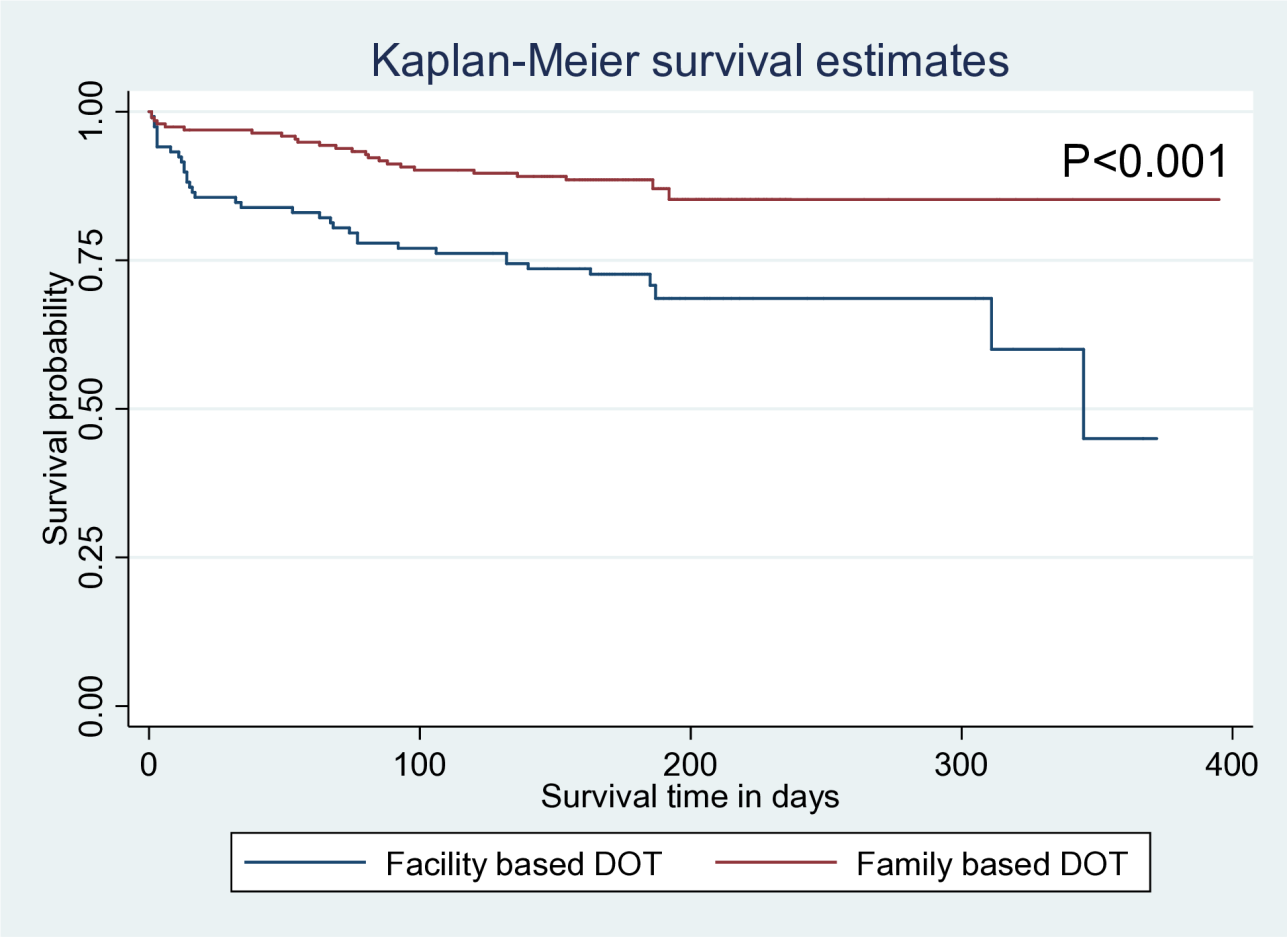
Kaplan–Meier survival curves comparing mortality among tuberculosis patients receiving facility-based (n = 120) versus family-based (n = 195) directly observed therapy (DOT) at Maamba General Hospital; log-rank test p-value < 0.001.

Fig 2 illustrates overall survival, the number of patients at risk, and the timing of deaths during follow-up. All 316 patients were followed for up to 12 months (360 days) after initiating tuberculosis treatment. Survival declined steadily over time, with 78.4% of patients alive at 6 months and 60.1% alive at 12 months, based on a total of 60 deaths recorded during follow-up. The majority of deaths (31/60; 51.7%) occurred within the first 60 days of treatment, while 40.0% (24/60) occurred within the first 30 days (1 month) (Table 2). Patients with bacteriologically confirmed TB and those managed under family-based directly observed therapy (DOT) had significantly better survival compared with clinically diagnosed patients (log-rank p = 0.006; Fig 3) and those receiving facility-based DOT (log-rank p < 0.001; Fig 4).

**Table 2:** Time to death for persons with TB on treatment.

Time interval (days)	Number at risk at start of interval	Deaths during interval, n (% of total deaths)	Censored during interval, n	Kaplan–Meier survival probability (%)	95% Confidence interval
0–30	316	24 (40.0)	5	92.2	88.8–94.8
30–60	287	7 (11.7)	0	90.1	86.2–92.9
60–90	280	13 (21.7)	1	86.2	81.9–89.6
90–120	267	4 (6.7)	2	84.9	80.5–88.5
120–150	261	5 (8.3)	11	83.3	78.6–87.0
150–180	245	2 (3.3)	105	82.4	77.6–86.2
180–210	138	4 (6.7)	88	78.9	73.0–83.7
210–240	46	0 (0.0)	25	78.9	73.0–83.7
240–270	21	0 (0.0)	4	78.9	73.0–83.7
270–300	17	0 (0.0)	1	78.9	73.0–83.7
300–330	16	1 (1.7)	7	72.5	57.3–83.1
330–360	8	1 (1.7)	4	60.5	32.9–79.7
360–390	3	0 (0.0)	2	60.5	32.9–79.7
390–420	1	0 (0.0)	1	60.5	32.9–79.7

Footnote:

Percentages in the “Deaths during interval” column were calculated as: (number of deaths occurring in the interval ÷ total number of deaths observed during follow-up [n = 60]) × 100.

Kaplan–Meier survival probabilities were estimated using S(t) = ∏ (1 − dᵢ/nᵢ), where dᵢ is the number of deaths and nᵢ is the number of persons at risk at the start of interval i.

Participants were censored due to loss to follow-up, transfer out, completion of treatment, or end of observation.

### Predictors of TB mortality

In the crude analysis, for each one-year increase in age, the risk of mortality increased by 2% (p = 0.015). Clinical diagnosis was associated with a significantly higher risk of mortality compared to bacteriological diagnosis (p = 0.011). For every 1-kg increase in weight at the start of treatment, the risk of mortality decreased by 5% (p = 0.002). Participants under a family-based DOT plan had a lower risk of mortality compared to those on a facility-based DOT plan (p < 0.001).

In the multivariable analysis, the risk of mortality increased by 1.7% for each one-year increase in age (p = 0.042). PLHIV had an increased risk of mortality compared to HIV-negative individuals (p = 0.039). Participants with a clinical diagnosis for TB had a 97% higher risk of mortality compared to those with a bacteriological diagnosis (p = 0.015). For every 1-kg increase in weight at the start of treatment, the risk of mortality decreased by 4% (p = 0.004). Participants under a family-based DOT plan had a lower risk of mortality compared to those on a facility-based DOT plan (p = 0.003) (see Table 3).

**Table 3:** Crude and adjusted analysis of factors associated with TB mortality.

Variable	cHR (95% Cl)	P-value	aHR (95%Cl)	P-value
**Age**	1.02 (1.00, 1.04)	**0.015**	1.017 (1.000, 1.033)	**0.042**
**Sex**				
**Male**	Ref		ref	
**Female**	0.75 (0.43, 1.31)	0.320	0.60 (0.32, 1.12)	0.107
**Employment**				
**Formal**	ref		ref	
**Informal**	0.65 (0.23, 1.79)	0.405	0.67 (0.20, 2.23)	0.509
**PLHIV**				
**No**	Ref		ref	
**Yes**	1.53 (0.90, 2.61)	0.116	1.84 (1.03, 3.29)	**0.039**
**Type of TB**				
**Disseminated**	Ref		Ref	
**Pulmonary**	1.38 (0.64, 3.01)	0.406	1.43 (0.53, 3.85)	0.471
**Diagnosis**				
**Bacteriological**	Ref		ref	
**Clinical**	1.97 (1.17, 3.31)	**0.011**	1.97 (1.14, 3.41)	**0.015**
**Weight at start of Treatment, *kg***	0.95 (0.93, 0.98)	**0.002**	0.96 (0.92, 0.99)	**0.004**
**DOT plan**				
**Observed Daily at Clinic**	Ref		ref	
**Observed daily by family**	0.37 (0.22, 0.62)	**< 0.001**	0.42 (0.24, 0.74)	**0.003**

Abbreviation:

**DOT: Direct observation therapy, cHR: crude Hazard ratio, aHR: adjusted Hazard ratio, PLHIV: People Living with HIV**

Footnote:

**N in the adjusted model denotes participants with complete data included in the multivariable Cox regression (n = 284). Adjusted hazard ratios (aHRs) with 95% confidence intervals were estimated using complete-case analysis.**

## Discussion

This study investigated TB-related mortality at Maamba General Hospital, focusing on time to death and its predictors. The findings revealed a TB mortality incidence of 19.0%, which is substantially higher than the national average of 6% and also higher than reports from other regions in Zambia, such as the Southern Province and Lusaka [6,7,13]. The observed disparity may be partially accounted for by variations in methodological approaches across studies. For instance, our study focused on the use of retrospective hospital-based data, which may capture more deaths than community-based surveillance. These findings emphasize the critical need for targeted interventions in our setting, where healthcare access and resources are often constrained.

The majority of the mortalities occurred in the first two months of the intensive treatment phase, specifically within the initial month. These findings align with earlier evidence from studies conducted in Zambia and Ethiopia [7,14]. This could be due to the fact that several individuals who died were clinically diagnosed with tuberculosis, and some may have had other differential diagnoses that mimic TB, potentially leading to misdiagnosis despite initiation of anti-tuberculosis therapy [15]. Additionally, this could be attributed to delays between testing and the initiation of treatment, as often observed in resource-limited settings, with patients frequently arriving at facilities when the illness has progressed to an advanced stage [16]. Enhancing community awareness, understanding, and demand for tuberculosis services is crucial. Robust study designs are needed to elucidate the health-seeking behaviors and care trajectories of individuals affected by tuberculosis in low- and middle-income countries.

This study found that increasing age was associated with higher mortality. These findings are consistent with a study that reported a 2.4% increase in mortality hazard per year of age [17]. Similarly, another study explained that individuals aged 65 and older face increased susceptibility to TB and higher mortality rates due to factors such as decreased lung function and immunosenescence [18]. Age-related increases in functional residual capacity, along with declines in lung elasticity and immunity, reduce the body’s ability to clear TB infections, increasing vulnerability and worsening outcomes [19]. Additionally, immunosenescence, a gradual decline in immune system function with age, leads to impaired innate and adaptive immune responses [18]. This includes reduced T-cell function, lower cytokine production, and decreased capacity to mount a robust immune defense against Mycobacterium tuberculosis [19]. These physiological changes make elderly individuals more vulnerable to severe disease progression and complications [19]. These findings underscore the importance of screening and implementing tuberculosis preventive therapy among elderly patients to reduce associated risks and improve survival outcomes.

This study found that tuberculosis-related mortality was higher among individuals living with HIV compared to those who were HIV-negative. Similar findings have been reported in studies from Tanzania (2020) and Ethiopia (2022), underscoring the persistent vulnerability of this population [20,21]. The association is biologically credible, as HIV-induced immunosuppression impairs the host’s ability to contain *Mycobacterium tuberculosis* [22]. In this cohort, individuals with HIV/TB co-infection had lower median CD4 counts than survivors, indicating weakened immune function. Although the difference was not statistically significant, the trend supports established evidence that reduced immune competence contributes to more severe disease progression and poorer treatment outcomes [22]. However, information on antiretroviral therapy (ART) use at TB treatment initiation and the timing of ART initiation was not consistently available in the routine clinical records, limiting our ability to adjust for these important factors. Consequently, residual confounding in the association between HIV status and mortality cannot be excluded and should be considered when interpreting these findings.

Our study also found that people with clinically diagnosed TB had a higher risk of dying compared to those with bacteriologically confirmed TB. Similar findings were reported in Ethiopia, South Africa, and Nigeria [23,24]. Asgedom et al. argued that in resource-limited settings, death among persons with clinically diagnosed TB may be due to incorrect diagnosis as differentiating PTB from other conditions such as pneumocystis jirovecii pneumonia can be very difficult in a routine clinical setting [25]. Comprehensive evaluation of persons with TB prior to treatment initiation and close monitoring of response to treatment may help detect undiagnosed conditions with similar presentation to tuberculosis [26]. Continuous mentorship can also help build service providers’ capacity and skills to differentiate TB from other related conditions, as well as support the formulation of guidelines in such cases [27].

This study found that individuals with TB on a facility based Directly Observed Treatment (DOT) plan was associated with increased risk of mortality compared to those on home-based DOT. This finding appears to contradict a Zambian study (2024), which showed no significant difference between facility and community [5]. A potential explanation for our finding is confounding by indication, where sicker patients or those lacking social support are more likely assigned to facility-based DOT. As we did not adjust for baseline disease severity, this may explain the observed association. These results should be interpreted cautiously and highlight the need for research that accounts for illness severity. Facility-based DOT programs may also require additional support, such as closer monitoring or tailored interventions, for higher-risk patients.

A unit increase in weight at the start of treatment was associated with a reduced risk of TB mortality, a finding that has also been reported in studies from Denmark (2020), Taiwan (2017), and Georgia (2024), where lower baseline weight or BMI was linked to significantly higher mortality during TB treatment [28–30]. Undernutrition has been shown to impair both innate and adaptive immunity, reducing the body’s ability to mount an effective response to *Mycobacterium tuberculosis* infection [31]. Undernourished individuals exhibit decreased production of pro-inflammatory cytokines, reduced T-cell function, and impaired macrophage activity, all of which are critical for controlling TB infection [31]. Therefore, there is a need to comprehensively conduct nutritional assessments and provide nutritional support in our setting, where such measures are often lacking. Implementing these interventions could lead to improved outcomes among patients diagnosed with TB.

This single-center study may have limited generalizability, and the use of routine programmatic data meant that several important factors associated with tuberculosis mortality such as comorbidities, socioeconomic status, behavioral risks, and detailed markers of disease severity (hospitalization at treatment initiation, chest X-ray findings, oxygen saturation, performance status, and bacillary load) were not available. Potential confounding by indication, particularly in the assignment of DOT type, cannot be excluded, as patients with more severe diseases may have been preferentially managed under facility-based DOT. Despite these limitations, the study provides important insights into tuberculosis mortality and its predictors in a rural setting.

## Conclusion

This study highlights high TB-related mortality in a rural health facility, especially among older adults, HIV-positive individuals, and those clinically diagnosed. Family-based DOT and higher weights were associated with reduced mortality. To address this, the study recommends strengthening opportunities for screening and the use of TB preventive therapy among elderly TB patients, providing nutritional assessment, strengthening family-based DOT, as well as improving the diagnostic capacity to differentiate conditions with similar presentations to tuberculosis.

## Supporting information

S1 File Strobe check list. (DOCX)

S2 File Minimal dataset. (XLSX)
