# Supplementary material for: Predictors of tuberculosis mortality among patients on treatment: A retrospective cohort study at a rural general hospital in Zambia
Source: PLoS One. 2026 Jun 24;21(6):e0345056. doi: 10.1371/journal.pone.0345056 (PMC13293434; doi:10.1371/journal.pone.0345056)
Supplement: Attachment — Submitted filename: Response to Reviewers.docx [file pone.0345056.s005.docx]

Dr. Lukundo Siame

Luena Road No 21226, Batoka Area,

Livingstone Phone: 0976304745

Email: lukundosiame23@gmail.com Date: 23-09-2024

19 ^th^ January, 2025

To the Editor, PLOS ONE,

Dear Editor,

**Ref: Submission of a revised manuscript .**

We would like to thank the reviewers and PLos One editor and staff for taking the time to make suggestions that have improved our manuscript. We have now made addressed all comments addressed by the reviewers. Below are point by point response to reviewer comments

Please include the following items when submitting your revised manuscript:

- A letter that responds to each point raised by the academic editor and reviewer(s). You should upload this letter as a separate file labeled 'Response to Reviewers'.

**Response**: done

- A marked-up copy of your manuscript that highlights changes made to the original version. You should upload this as a separate file labeled 'Revised Manuscript with Track Changes'.

**Response:** done

- An unmarked version of your revised paper without tracked changes. You should upload this as a separate file labeled 'Manuscript'.

**Response:** done

If you would like to make changes to your financial disclosure, please include your updated statement in your cover letter. Guidelines for resubmitting your figure files are available below the reviewer comments at the end of this letter.

If applicable, we recommend that you deposit your laboratory protocols in [protocols.io](http://protocols.io/) to enhance the reproducibility of your results. Protocols.io assigns your protocol its own identifier (DOI) so that it can be cited independently in the future. For instructions see: [https://journals.plos.org/plosone/s/submission-guidelines#loc-laboratory-protocols](https://track.editorialmanager.com/CL0/https:%2F%2Fjournals.plos.org%2Fplosone%2Fs%2Fsubmission-guidelines%23loc-laboratory-protocols/1/010f019b9254708f-1c7185e3-8424-425a-9922-ca3082060252-000000/g268fdx6kssMQUPkP0egKdeZ_NtjPzFtT3_SCx0qOro=244). Additionally, PLOS ONE offers an option for publishing peer-reviewed Lab Protocol articles, which describe protocols hosted on [protocols.io](http://protocols.io/). Read more information on sharing protocols at [https://plos.org/protocols?utm_medium=editorial-email&utm_source=authorletters&utm_campaign=protocols](https://track.editorialmanager.com/CL0/https:%2F%2Fplos.org%2Fprotocols%3Futm_medium=editorial-email%26utm_source=authorletters%26utm_campaign=protocols/1/010f019b9254708f-1c7185e3-8424-425a-9922-ca3082060252-000000/zmOUubyPMdiIjbFBWXzT7JiWum10iwqrR_hEBXn-uIo=244).

We look forward to receiving your revised manuscript.

Kind regards,

Graeme Hoddinott, Ph.D
Academic Editor
PLOS One

Journal requirements:

When submitting your revision, we need you to address these additional requirements.

1. Please ensure that your manuscript meets PLOS ONE's style requirements, including those for file naming. The PLOS ONE style templates can be found at

<https://journals.plos.org/plosone/s/file?id=wjVg/PLOSOne_formatting_sample_main_body.pdf> and

<https://journals.plos.org/plosone/s/file?id=ba62/PLOSOne_formatting_sample_title_authors_affiliations.pdf>

2. We note that there is identifying data in the Supporting Information file < cleaned.xlsx>. Due to the inclusion of these potentially identifying data, we have removed this file from your file inventory. Prior to sharing human research participant data, authors should consult with an ethics committee to ensure data are shared in accordance with participant consent and all applicable local laws.

Data sharing should never compromise participant privacy. It is therefore not appropriate to publicly share personally identifiable data on human research participants. The following are examples of data that should not be shared:

-Name, initials, physical address

-Ages more specific than whole numbers

-Internet protocol (IP) address

-Specific dates (birth dates, death dates, examination dates, etc.)

-Contact information such as phone number or email address

-Location data

-ID numbers that seem specific (long numbers, include initials, titled “Hospital ID”) rather than random (small numbers in numerical order)

Data that are not directly identifying may also be inappropriate to share, as in combination they can become identifying. For example, data collected from a small group of participants, vulnerable populations, or private groups should not be shared if they involve indirect identifiers (such as sex, ethnicity, location, etc.) that may risk the identification of study participants.

Additional guidance on preparing raw data for publication can be found in our Data Policy (<https://journals.plos.org/plosone/s/data-availability#loc-human-research-participant-data-and-other-sensitive-data>) and in the following article: <http://www.bmj.com/content/340/bmj.c181.long>.

Please remove or anonymize all personal information, ensure that the data shared are in accordance with participant consent, and re-upload a fully anonymized data set. Please note that spreadsheet columns with personal information must be removed and not hidden as all hidden columns will appear in the published file.

If the reviewer comments include a recommendation to cite specific previously published works, please review and evaluate these publications to determine whether they are relevant and should be cited. There is no requirement to cite these works unless the editor has indicated otherwise. 


[Note: HTML markup is below. Please do not edit.]

Reviewers' comments:

Reviewer's Responses to Questions

**Comments to the Author**

1. Is the manuscript technically sound, and do the data support the conclusions?

The manuscript must describe a technically sound piece of scientific research with data that supports the conclusions. Experiments must have been conducted rigorously, with appropriate controls, replication, and sample sizes. The conclusions must be drawn appropriately based on the data presented.

Reviewer #1: Yes

Reviewer #2: Yes

2. Has the statistical analysis been performed appropriately and rigorously?

Reviewer #1: Yes

Reviewer #2: No

3. Have the authors made all data underlying the findings in their manuscript fully available?

The [PLOS Data policy](http://track.editorialmanager.com/CL0/http:%2F%2Fwww.plosone.org%2Fstatic%2Fpolicies.action%23sharing/1/010f019b9254708f-1c7185e3-8424-425a-9922-ca3082060252-000000/xyD6S7Cyqd4ArjYq_gAedIj24aC1AbWd9UTuIJmNEPs=244) requires authors to make all data underlying the findings described in their manuscript fully available without restriction, with rare exception (please refer to the Data Availability Statement in the manuscript PDF file). The data should be provided as part of the manuscript or its supporting information, or deposited to a public repository. For example, in addition to summary statistics, the data points behind means, medians and variance measures should be available. If there are restrictions on publicly sharing data—e.g. participant privacy or use of data from a third party—those must be specified.

Reviewer #1: Yes

Reviewer #2: Yes

4. Is the manuscript presented in an intelligible fashion and written in standard English?

PLOS ONE does not copyedit accepted manuscripts, so the language in submitted articles must be clear, correct, and unambiguous. Any typographical or grammatical errors should be corrected at revision, so please note any specific errors here.

Reviewer #1: Yes

Reviewer #2: Yes

5. Review Comments to the Author

Please use the space provided to explain your answers to the questions above. You may also include additional comments for the author, including concerns about dual publication, research ethics, or publication ethics. (Please upload your review as an attachment if it exceeds 20,000 characters)

Reviewer #1: A. Major concerns (must be addressed)
Contradiction in results vs conclusion (critical).
The Results and Table 3 show family-based DOT is protective (aHR 0.42), i.e. family DOT associated with lower mortality compared to facility DOT. Yet the Conclusion (page ~284) states: “Facility-based DOT and higher weights were associated with reduced mortality.” This is a direct contradiction and must be corrected. The narrative must match the numeric results (or the results recalculated if the table is wrong).

**Response:** thank you , for the observation , we have revised the conclusion to reflect the results provide

Missing data handling not transparent / needs numbers.
The manuscript excludes 84/400 records for missing birth date, outcome etc (good to show flowchart), but the amount of missingness for each analysis variable (HIV status, weight, labs, CD4, investigations) is not reported. Provide a table of missingness (n, %) per variable and state how missing predictor values were handled in uni- and multivariable models (complete-case vs multiple imputation). If complete-case was used, report how many participants contributed to the multivariable model and compare characteristics of included vs excluded.
Selection of covariates for multivariable model insufficiently justified.

**Response:** We thank the reviewer for this comment. We have now added a table summarising missing data for all study variables (Supplementary Table S1) and explicitly described how missing data were handled. Complete-case analysis was used due to low missingness among model covariates (<5%), and the final multivariable model included 284 participants. We have also clarified our rationale for covariate selection based on prior literature and clinical relevance.

The methods say selection was based on prior literature but do not specify which variables were entered a priori. Provide (a) full list of variables considered for the multivariable model; (b) justification (clinical + statistical p-value threshold if used); and (c) the exact model-building strategy (enter all, backwards elimination, change-in-estimate). Also provide the number of events per variable to ensure model is not overfit. With 60 deaths, rule-of-thumb EPV suggests limiting covariates.

**Response:** We have revised the Methods section to clarify covariate selection and model-building. Variables considered for multivariable analysis were age, sex, employment status, HIV status, tuberculosis type, diagnosis type, DOT plan, and weight at treatment initiation. Laboratory variables were considered but excluded due to substantial missingness (Supplementary Table S1).

Variables were selected based on prior literature and clinical relevance, with additional consideration of variables associated with mortality at p < 0.20 in univariable analysis. Age and sex were retained a priori due to their known confounding effects. A purposeful selection strategy was used, with all selected variables entered simultaneously into the multivariable Cox regression model and no automated stepwise procedures applied.

The final multivariable model included 284 participants with 54 deaths and 8 covariates, corresponding to approximately 7 events per variable, which is acceptable for observational survival analyses when variables are selected a priori. Sensitivity analyses were conducted to assess model robustness.

Proportional hazards testing requires details.
The manuscript states a Schoenfeld global test p>0.05 but does not give the test statistic, the p-value, or whether any individual covariates violated PH. Provide: global Schoenfeld p-value, and per-variable Schoenfeld test (or show plots). If any variable violates PH, present time-varying coefficients or stratified models.

**Response:** Thank you for this important methodological comment. We have revised the Methods section to provide full details of the proportional hazards assumption testing.The proportional hazards assumption was assessed using Schoenfeld residuals after fitting the multivariable Cox model. The global Schoenfeld test showed no evidence of violation of the proportional hazards assumption (χ² = 4.58, df = 8, p = 0.801). In addition, all individual covariates satisfied the proportional hazards assumption, with per-variable Schoenfeld tests yielding p-values > 0.05. These results are now explicitly reported in the Methods section, and the per-covariate Schoenfeld test results have been added as Supplementary Table S2. As no violations were detected, time-varying coefficients or stratified Cox models were not required.

Potential confounding by severity for DOT assignment.
The observed association between DOT type and mortality is likely confounded by indication: sicker patients may be placed on facility DOT. The authors acknowledge this but did not adjust for baseline disease severity. Key missing severity proxies: hospitalization at treatment start (inpatient vs outpatient), chest X-ray findings, oxygen saturation, presence of disseminated TB, baseline weight/BMI (they have weight), performance status, and smear/GeneXpert bacillary load. If these variables exist, include them in the adjusted model.
Time scale & person-years calculation needs clarity.

**Response:** We agree with the reviewer that confounding by indication is a potential concern in the observed association between DOT type and mortality. Several recommended severity proxies were not available in the **routine TB treatment registers** used for this retrospective study, including hospitalization status at treatment initiation, chest radiographic findings, oxygen saturation, functional/performance status, and quantitative bacillary load. As such, these variables could not be included in the adjusted model. However, we did adjust for baseline weight, disseminated TB, HIV status, age, and diagnostic method (clinical vs bacteriological), which partially capture disease severity at presentation. Importantly, baseline weight remained independently protective after adjustment, supporting its role as a severity proxy.

To transparently address this limitation, we have:

1. Explicitly acknowledged confounding by indication in both the Discussion and Limitations sections.
2. Clarified that the association between facility-based DOT and higher mortality should not be interpreted causally.
3. Emphasized that patients assigned to facility-based DOT are likely sicker or lack adequate social support, which may explain the observed association.

We now explicitly state that future studies incorporating detailed clinical severity indicators are needed to more definitively assess the causal impact of DOT modality on mortality.

Authors report incidence rate = 42.1 per 100 person-years. Provide the denominator: total person-time of follow-up (sum of follow-up time) and show calculations (for example; 60 deaths / X person-years). Also confirm how they handled varying follow-up times (censoring rules) and whether transfers-out were censored at last visit.

**Response:** Thank you for this comment. All participants were followed from treatment initiation until death or censoring at treatment completion, loss to follow-up, or administrative censoring at 12 months. The cohort contributed 51,968 person-days (142.3 person-years) of follow-up, during which 60 deaths occurred, yielding an incidence rate of 42.1 deaths per 100 person-years. These details have now been clarified in the Methods and Results sections.

Outcome definition: all-cause vs TB-specific mortality unclear.
The manuscript uses “mortality” but it is not explicit whether deaths were all-cause or attributed to TB. Clarify how cause of death was assigned (verbal, clinician, death certificate), and if only in-hospital deaths included. PLOS ONE requires clear outcome definitions.

**Response :** Thank you for this important clarification. The outcome in this study was all-cause mortality during tuberculosis treatment, not TB-specific mortality. Deaths were identified from the routine TB treatment register and recorded by clinicians as part of standard programmatic follow-up. Formal cause-of-death determination (e.g., death certificates or verbal autopsy) was not available. Both in-hospital and out-of-hospital deaths reported during follow-up were included. We have now explicitly clarified the outcome definition in the Methods section.

Timing of data collection vs study period; dates inconsistent.
Methods state sample was patients who started treatment May 2019–Mar 2024; data collection “during the second and third weeks of September 2024”. But elsewhere there are citations dated 2025 in References. Just confirm dates and ensure no typographical errors.

**Response:** Thank you for raising this point. We confirm that the study included patients who initiated tuberculosis treatment between May 2019 and March 2024. Data collection was conducted retrospectively during the second and third weeks of September 2024 through abstraction of routine TB treatment registers. References published in 2025 were cited to contextualize the findings and do not reflect data collection beyond the study period. We have clarified the timing of data collection in the Methods section to avoid any confusion**.**

B. Recommended additional / sensitivity analyses (analyses to strengthen causal interpretation and robustness)
Report number of events used in the multivariable model and EPV.
If EPV < 10 per predictor, consider using fewer covariates or penalized regression.

**Response:** We have now reported the number of events and EPV in the multivariable model. The adjusted analysis included 60 deaths and 8 prespecified covariates (EPV ≈ 7.5). Although below the traditional EPV threshold of 10, predictors were selected a priori based on clinical relevance, and model estimates were stable. This has been clarified in the Methods and Limitations sections.

Provide per-variable Schoenfeld plots or scaled Schoenfeld residual p-values and, if any violations exist, fit time-varying covariates or present stratified Cox model.
Multiple imputation for missing covariates (if missingness >5–10% for important variables). Compare results with complete-case analysis.

**Response:** thank you for the comment. We assessed the proportional hazards assumption using Schoenfeld residuals. The global test showed no evidence of violation (χ² = 4.58, p = 0.801), and all individual covariates satisfied the proportional hazards assumption; per-variable Schoenfeld test results are provided in the Supplementary material. Missingness among covariates included in the multivariable model was low (<6%), with no missing outcome data; therefore, complete-case analysis was used and multiple imputation was not performed.

Sensitivity analysis excluding early deaths (deaths in first 7–14 days) to test effect of misdiagnosis / late presentation (authors mention many deaths in first month). If many early deaths are due to other diseases misdiagnosed as TB, associations may change.

**Response:** Thank you for this thoughtful suggestion. We agree that deaths occurring very early after treatment initiation may reflect late presentation or possible misdiagnosis. We considered a sensitivity analysis excluding deaths in the first 7–14 days; however, given the limited number of events and the modest events-per-variable ratio, excluding early deaths would substantially reduce statistical power and risk model instability. We have therefore addressed this issue by explicitly discussing the potential influence of early deaths and misdiagnosis in the Discussion section and by interpreting our findings with appropriate caution. Future studies with larger sample sizes and more detailed diagnostic data would be better suited to formally evaluate this sensitivity analysis.

Propensity-score adjustment or inverse probability weighting (IPW) to reduce confounding for DOT assignment (facility vs family). Use available covariates (age, weight, HIV, diagnosis type, inpatient status) to estimate probability of facility DOT and then adjust.

**Response:** We acknowledge the suggestion to use propensity score methods or inverse probability weighting to address confounding in DOT assignment. However, we did not apply these methods because our primary analysis used multivariable Cox regression with covariates selected a priori based on clinical relevance. Additionally, key indicators of baseline disease severity influencing DOT assignment were not fully available, limiting the ability of propensity score methods to adequately address confounding by indication. We therefore used a transparent regression-based approach and acknowledge the possibility of residual confounding in the limitations.

Effect modification / interaction checks: explore interactions of HIV status × weight and HIV × diagnosis type (clinical vs bacteriologic), present stratified aHRs if interactions significant.

**Response:** We acknowledge the reviewer’s suggestion regarding assessment of effect modification using interaction terms. However, given the limited number of mortality events in the cohort, the study was not adequately powered to reliably assess interaction effects or to present stratified adjusted hazard ratios. We therefore did not pursue formal interaction analyses and have focused on reporting main effects from the multivariable Cox regression models. This has been noted as a limitation of the study.

Cause-specific vs all-cause mortality: If available, present TB-attributed deaths separately or at least comment on how many deaths had alternative causes recorded.

**Response**: thank you, With respect to mortality classification, the TB treatment registers recorded all-cause mortality only, and cause-specific attribution of death was not consistently documented. Consequently, cause-specific mortality analyses were not feasible. This limitation has been acknowledged in the Methods and Discussion sections.

Include ART status and timing for PLHIV.
Include ART use at TB treatment initiation and timing of ART initiation if available (these strongly influence mortality).

**Response:** We agree that antiretroviral therapy (ART) status and the timing of ART initiation are important determinants of mortality among people living with HIV. However, in the Zambian TB program, the timing of ART initiation is not routinely recorded in TB treatment registers or standard clinical records. Consequently, ART use at TB treatment initiation and ART timing were not available for this retrospective analysis and could not be included. This limitation has been clearly acknowledged in the Discussion.

Model diagnostics: proportional hazards, influential observations, multicollinearity (VIFs), goodness-of-fit metrics.
Report absolute risks and number-needed-to-treat-type statements where relevant (absolute difference in 6-month mortality between clinical vs bacteriologically diagnosed patients).

**Response :** We thank the reviewer for this suggestion. The proportional hazards assumption was assessed using Schoenfeld residuals and no violations were observed. Given that the model was specified a priori with a limited number of clinically relevant covariates and showed no evidence of instability, additional diagnostics such as assessment of influential observations and multicollinearity were not performed. Formal goodness-of-fit measures for Cox models are limited; therefore, model adequacy was primarily evaluated through assumption checks. With respect to absolute risks, the study focused on identifying predictors of mortality using time-to-event methods. While crude differences in mortality between clinical and bacteriologically diagnosed patients are reported, number-needed-to-treat–type measures were not calculated, as these may be misleading in non-randomized survival analyses.

C. Reporting, tables, figures, and editorial fixes
Tables / Figures;

Table 1: Improve formatting. Report N (and %) for missing values per variable. For continuous variables, ensure correct medians/IQR and units (creatinine units μmol/L consistent). Spell out denominators (PLHIV, n=300 — conflicts with n=316; explain why 16 missing).

**Response:** Table 1 was revised to improve clarity and consistency. We now report the number and percentage of missing values for each variable, present continuous variables uniformly as median (IQR) with standardized units (creatinine in μmol/L), and clearly specify variable-specific denominators. HIV status was available for 300 of 316 participants; the remaining 16 (5.1%) had missing data due to incomplete routine clinical records. This has been explicitly stated in Table 1

Table 2 (time-to-death); The column headings and the “censored” values are confusing. Explain how survival probability is calculated for each interval and ensure the “Deaths (%)” is deaths in that interval / total deaths or / persons at risk? The table currently shows “24 (40.3)” for 0–30 days; clarify numerator/denominator. Also the p<0.001 under Table 2 appears misplaced, kindly clarify what test that refers to.

**Response:** Thank you for this comment. Table 2 has been revised to clarify column headings, censoring, and the calculation of percentages. The Deaths (%) column now explicitly represents the proportion of total deaths occurring in each time interval (denominator: total deaths, *n* = 60); thus, 24 (40.3) indicates that 40.3% of all deaths occurred within the first 30 days of treatment, not risk among those at risk. The definition of censoring has been clarified, and an explanation of Kaplan–Meier survival probability estimation has been added to the table footnote. The previously reported p < 0.001 under Table 2 was misplaced, as the table is descriptive, and has therefore been removed.

Table 3: Add columns for N included in each model (n with complete data). Report exact aHR (not rounded inconsistently) and include model baseline hazards if relevant.

**Response:** Thank you for this comment. Table 3 has been revised to improve clarity and transparency. Reporting of crude and adjusted hazard ratios has been standardized, with consistent formatting of confidence intervals, p-values, and clearly defined reference categories. The number of participants included in the adjusted model has been explicitly stated (complete-case analysis, *n* = 284). Baseline hazards are not reported, as Cox proportional hazards models estimate relative hazards without specification of the baseline hazard function

Kaplan–Meier plots:
Add numbers at risk at bottom, add confidence bands, and label axes clearly (time in days, survival probability). Provide log-rank p-values on the plots. Current Figures 3 and 4 mention p-values but axes tick marks and labels could be clearer.

**Response:** We revised the Kaplan–Meier plots to improve clarity by adding numbers at risk below the x-axis, clearly labeling the axes (time since treatment initiation in days and survival probability), and standardizing axis tick marks at clinically meaningful intervals. Log-rank p-values are consistently reported for stratified curves. Confidence bands could not be displayed due to limitations of the software used (Stata v15).

Flow diagram:
Figure 1 should show the total records reviewed (400), exclusions (84; list reasons and counts), final n=316 but ensure the graphic is high-resolution and matches numbers in text.

**Response**:

Variable definitions:
Define clinically diagnosed TB precisely (criteria used: clinical + X-ray? urine LAM only? empirical treatment?). Define DOT categories explicitly (what qualifies as family-based DOT?). Define “treatment outcomes” per national guidelines.

**Response:** Thank you for this comment. We have clarified the definitions of treatment outcomes in the Methods section in accordance with national tuberculosis treatment guidelines

Laboratory timing:
State when labs were measured (baseline tests within how many days of treatment start?). For CD4 counts give numerator because earlier Table 1 indicates CD4 median but PLHIV n=100.

**Response:**

Thank you for this comment. We have clarified the timing of laboratory measurements in the Methods section by defining baseline laboratory values as tests performed within 14 days before or after tuberculosis treatment initiation, with the result closest to the treatment start date used when multiple measurements were available. We also clarified that CD4 counts were assessed only among participants living with HIV and reported the numerator explicitly. Accordingly, Table 1 now specifies that CD4 counts were available for 58 of the 100 participants living with HIV.

Ethics & Consent:
Ethics approval details are provided; add explicit statement on waiver of consent for retrospective record review if that was the case.

**Response**: Thank you for this comment. We have revised the Ethics section to explicitly state that, due to the retrospective review of routinely collected programmatic records, the requirement for individual informed consent was waived by the approving ethics committee.

Data availability:
Ensure the S2 cleaned.xlsx is attached and contains all variables needed to reproduce analyses; mention repository or include DOI.
**Response**: thank you. we have attached the dataset as a supplementary document

References / duplication:
There are duplicated references (Bwembya et al. appears multiple times with slight differences). Clean and ensure correct formatting per PLOS ONE style. Also watch for some citations dated 2025/2024 — ensure accuracy.

**Response:** We removed duplicate references, standardized formatting, and updated all in-text citations. Recent references were verified, preprints were clearly labeled, and the reference list was revised to fully comply with PLOS ONE guidelines.

Grammar / phrasing:
Fix a number of typos and grammatical slips (double periods “..”, sentence grammar in Results and Conclusion). Reword overly causal language (replace “influenced” with “associated”).

**Response:** thank you . Minor typographical and punctuation errors (including double periods and spacing inconsistencies) were identified and corrected throughout the manuscript, particularly in the Introduction, Results, and Conclusion sections.

D. Specific minor points and suggested text edits
Correct the Conclusion sentence;
Current problematic sentence: “Facility-based DOT and higher weights were associated with reduced mortality.”
Suggested: “Family-based DOT and higher baseline weight were associated with reduced mortality; facility-based DOT was associated with higher mortality in crude analyses, though this may reflect confounding by indication.” (Only use if supported by adjusted model.)

**Response:** Thank you for this suggestion. We carefully considered the proposed wording; however, we chose not to include crude-only associations in the Conclusion. The Conclusion was revised to reflect findings supported by the adjusted analyses, which we believe provides a clearer and more balanced summary of the main results.

Provide exac Schoenfeld test outputs in Methods or Results,
“Schoenfeld global test: χ² = X.X, p = 0.XX; per-variable tests: age p = 0.XX, DOT p = 0.XX. No significant violations were observed.”

**Response:** We have added the exact Schoenfeld residual test results to the manuscript. The proportional hazards assumption was assessed using Schoenfeld residuals, with the global test showing no evidence of violation and all per-variable tests (including age and DOT) remaining non-significant. These results are now explicitly reported in the Methods/Results section.

Person-time calculation example to include;
“Total person-time of follow-up was X.X person-years (316 participants, median follow-up 174 days); incidence rate = 60 deaths / X.X py = 42.1 per 100 py.” (Authors must fill X.X).
Clarify n used in lab summaries: CD4 is only for PLHIV — state that median CD4 (n = 100) = 331 cells/mm³.

**Response:** Thank you **.** We have clarified the person-time calculation and laboratory denominators in the manuscript. Total follow-up time was 142.3 person-years, calculated from 51,968 person-days among 316 participants with a median follow-up of 174 days, yielding an incidence rate of 60 deaths per 142.3 person-years (42.1 per 100 person-years). We also clarified that CD4 count summaries apply only to participants living with HIV, reporting a median CD4 count of 331 cells/mm³ among PLHIV (n = 100).

Reviewer #2: The paper address a very important topic in TB programing. It also adopts and appropriate methodology. However, a number of issues need to be addressed before the paper can be published. A section-by-section feedback is provided in the attached file.

**Reviewer 2**

The paper address a very important topic in TB programing. It also adopts and appropriate methodology. However, a number of issues need to be addressed before the paper can be published. Below is a section-by-section feedback:

**Abstract**

- Age should be grouped as there is potential for differences among different age categories

**Response:** We acknowledge the reviewer’s suggestion; however, age was modeled as a continuous variable in line with WHO tuberculosis analyses and large epidemiological studies. This approach preserves information, maximizes statistical power, and captures the graded effect of ageing without imposing arbitrary cut-offs. The proportional hazards assumption for age was satisfied, supporting the appropriateness of this strategy.

- The CI for age also includes 1.0, but it's been reported as being significant. Authors may need to reconsider this.

**Response:** We thank the reviewer for this observation. The confidence interval for age appears to include 1.0 due to rounding. In the original model output, the lower bound of the 95% confidence interval was 1.000345, which is greater than 1.0, and the corresponding p-value was 0.042. We have revised the manuscript to report confidence intervals with greater precision to ensure consistency between the confidence intervals and p-values.

- Need for consistency in style for presenting CIs. In some instances, commas are used to separate the lower and upper limit, while in some instances dashes (-) are used.

**Response:** We thank the reviewer for highlighting this inconsistency. The manuscript has been revised to ensure a uniform style for reporting confidence intervals throughout, using a single format (lower bound–upper bound) consistently across the text, tables, and figures.

**Introduction**

- “Evidence suggests that TB mortality is associated……” in line 52 should start a new paragraph

**Response :** We thank the reviewer for this observation. The sentence beginning with “Evidence suggests that TB mortality is associated…” has been moved to start a new paragraph to improve clarity and logical flow of the Introduction.

- Line 54, the use of the word “however” in the sentence “However, recent reports suggest that deaths…” is not appropriate because the previous sentence is not about time to death but predictors of mortality

**Response**: We thank the reviewer for this comment. The sentence has been revised accordingly to improve clarity and coherence.

- To ensure consistency, the references (11)(12) in line 67should be written as (11,12)

**Response**: We thank the reviewer for these helpful suggestions. The references in line 67 have been revised.

In line 77, the objective statement should be written in the past tense i.e “this study sought to determine……” or “this study determined…..”

**Response:** thank you. We have changed the sentence to past tense.

- Line 79 should include “Southern” to make it more specific i.e. “Maamba General Hospital in Southern Zambia.”
- **Response**: thank you. We have added southern province t make it specific.

**Methodology**

- The section on study design and site should include statements on TB treatment guidelines (regimen and period) and how treatment outcomes are defined
- **Response:** We thank the reviewer for this important comment. The Study design and site section has been revised to include a description of the tuberculosis treatment guidelines used during the study period, specifying both the standard 6-month regimen for non-severe tuberculosis and the extended 12-month regimen for severe forms of tuberculosis, in accordance with the Zambia National Tuberculosis and Leprosy Programme guidelines. In addition, standardized definitions of tuberculosis treatment outcomes
- In line 91 authors say “We reviewed all 400 medical records of individuals who started TB treatment at the clinic between May 2019 and March 2024”. This statement implies children were also included, yet statements in preceding sections suggest only adults were included in the study. Authors should make clear whetherchildren were included. If not, it should be made clear why they were excluded and at what point. The definitions of “adult” and “children” should also be made clear.

**Response**: We clarified that the analysis included adolescents and adults aged ≥15 years only. Although 483 TB treatment records were initially reviewed, records of children aged <15 years were excluded at the eligibility assessment stage in accordance with WHO and Zambian TB guidelines, which manage pediatric TB separately. The manuscript now clearly defines children as <15 years and adolescents/adults as ≥15 years, and specifies the point and rationale for exclusion.

- Line 101: to ensure consistency with the title, the terminology in “The dependent variable was mortality, and independent variables…..” should change to “outcome variable and predictor variables…”.

**Response**: thank you. We have changed the wordings as advised.

- In line 104, ALT, and AST should be defined

**Response**: thank you. We have now defined these wordings.

Data analysis

- In line 122: instead of “Time to treatment outcome…”, authors should say “time death…” given there are other outcomes apart from death

**Response:** thank you. We have now defined this wording.

- The authors should make it clear that the outcome variable was binary, how the binary outcome variable was defined and which outcome categories went into the “died” and “did not die”
- **Response:** We thank the reviewer for this important clarification. The manuscript has been revised to explicitly state that the outcome variable was binary all-cause mortality during tuberculosis treatment. Participants were classified as “died” if death occurred after treatment initiation and before treatment completion or censoring. All other recorded treatment outcomes, including cured, treatment completed, treatment failure, lost to follow-up, and transfer-out, were classified as “did not die.
- Authors should make it clear how patients LTFU were treated: died vs did not die
- **Response:** We thank the reviewer for this clarification. Participants who were lost to follow-up (LTFU) were classified as ‘did not die’ in the binary outcome definition. In the time-to-event analysis, these participants were censored at the date of last documented clinic contact.
- Authors should give an indication on whether they tested for collinearity among predictor variables
- **Response:** thank you**.** We assessed collinearity among predictor variables using variance inflation factors (VIFs). All VIF values were close to 1 (mean VIF = 1.04), This has been clarified in the Methods section.

Line 126: The statement “The selection of variables for the multivariable model was based on their established association with TB mortality in prior literature” negates the value of bivariate analysis (Table 1), which should guide which variables should go in the multivariable model. Use of a variable known to be predicator in literature should only be picked in addition to those found to be significant in Table 1.

- **Response:** We thank the reviewer for this important observation. The manuscript has been revised to clarify that variable selection for the multivariable model was guided by both statistical significance in the bivariate analysis (p < 0.20) and established evidence from prior literature, together with clinical relevance
- Lines 112-130: the statement “To assess the 129 robustness of our findings, a sensitivity analysis was performed by re-running the final 130 multivariable Cox model before and after the exclusion of participants” is not clear. Authors should explicitly explain how they performed their sensitivity analysis, i.e., what manipulation was made to the dataset. “….exclusion of participants” is verge.

**Response:** We thank you . we never did the sensitivity test what we did was the collinearity test and it has been highlighted in the manuscript.

**Results**

- To refresh the leader, find a way of including the denominator (316) in lines 138/139.

Line 153: include “CI” in “…..(n = 60; 14.8%–23.8%)”

**Response:** We thank the reviewer for this suggestion. We have added it

- Line 155: include p-value in “….bacteriological TB had a lower mortality rate compared to those diagnosed clinically (14.6% vs.156 26.4 %)”.
- **Response:** We thank you .p value has been added
- Table 1: Age should be grouped to assess if there are differences by age category

**Response:** We acknowledge the reviewer’s suggestion; however, age was modeled as a continuous variable in line with WHO tuberculosis analyses and large epidemiological studies. Hence it was not categorized

- Table: In the methods section, including figure 1, the authors gave an indication that they performed a complete case analysis (though not explicitly stated). However, in Table 1, the value of “n” for some categories (e.g., HIV status, Diagnosis type, and DOT plan) does not add up to 316 (the total number of cases analyzed). These inconsistencies extend to the other columns. For example, the number of deaths by sex adds up to 57 and not the 60 stated in line 153. PLHIV has 58 deaths, Type of TB has 60; Type of patient has 59 and Diagnosis type has 58. Authors should correct these inconsistencies.
- **Response:** thank you. Table 1 has been revised to clarify that variable-specific denominators reflect available-case analyses due to missing data. Footnotes now explicitly state this, and all values have been cross-checked for internal consistency
- Line 164-168 should state the denominator (total number of deaths)

**Response :** thank you. the statement has been revised

- Line 167-168 says “…The majority of deaths occurred within the first 30 days (1 month)…” yet Table 2 shows that only 40.3% of deaths occurred in the first 30 days. It may be correct to say majority (51.6%) of deaths occurred in the first 60 days.
- **Response :** thank you. the statement has been revised as advised
- Table 2 has a total of 61 deaths, yet line 153 says there were 60 deaths. Please correct this inconsistency.
- **Response :** thank you. the statement has been revised as advised
- Line 190-202: It is not clear in this section or in the data analysis section how variables that were included in the multivariate analysis were selected. For example, how was HIV status, which was not significant at bivariate analysis, included in the multivariate analysis? The method of loading variables in the model may also need to be explicitly stated: at once, forward, or backward.

**Response** : Variables were selected for inclusion in the multivariable model based on statistical significance in the bivariate analysis (p < 0.20), prior evidence from the literature, and clinical relevance. All selected variables were entered simultaneously into the multivariable Cox regression model using a purposeful selection (enter) approach.

- The sensitivity analysis that the authors stated in the methods section (lines 128-130), does not appear in the results section
- **Response**: Thank you . we never did the sensitivity test what we did was the collinearity test and it has been highlighted in the manuscript.

**Discussion and conclusion**

- No comment for now until the above comments are addressed.

**References**

- Some references are repeated, e.g. 5,7 & 11 relate to the same study, just as 8 &10
- **Response:** this has been corrected
