# Supplementary material for: Predictors of tuberculosis mortality among patients on treatment: A retrospective cohort study at a rural general hospital in Zambia
Source: PLoS One. 2026 Jun 24;21(6):e0345056. doi: 10.1371/journal.pone.0345056 (PMC13293434; doi:10.1371/journal.pone.0345056)
Supplement: Attachment — Submitted filename: Reviewer Comments_Josphat Bwembya.docx [file pone.0345056.s003.docx]

The paper address a very important topic in TB programing. It also adopts and appropriate methodology. However, a number of issues need to be addressed before the paper can be published. Below is a section-by-section feedback:

**Abstract**

- Age should be grouped as there is potential for differences among different age categories
- The CI for age also includes 1.0, but it's been reported as being significant. Authors may need to reconsider this.
- Need for consistency in style for presenting CIs. In some instances, commas are used to separate the lower and upper limit, while in some instances dashes (-) are used.

**Introduction**

- “Evidence suggests that TB mortality is associated……” in line 52 should start a new paragraph
- Line 54, the use of the word “however” in the sentence “However, recent reports suggest that deaths…” is not appropriate because the previous sentence is not about time to death but predictors of mortality
- To ensure consistency, the references (11)(12) in line 67should be written as (11,12)
- In line 77, the objective statement should be written in the past tense i.e “this study sought to determine……” or “this study determined…..”
- Line 79 should include “Southern” to make it more specific i.e. “Maamba General Hospital in Southern Zambia.”

**Methodology**

- The section on study design and site should include statements on TB treatment guidelines (regimen and period) and how treatment outcomes are defined
- In line 91 authors say “We reviewed all 400 medical records of individuals who started TB treatment at the clinic between May 2019 and March 2024”. This statement implies children were also included, yet statements in preceding sections suggest only adults were included in the study. Authors should make clear whether children were included. If not, it should be made clear why they were excluded and at what point. The definitions of “adult” and “children” should also be made clear.
- Line 101: to ensure consistency with the title, the terminology in “The dependent variable was mortality, and independent variables…..” should change to “outcome variable and predictor variables…”.
- In line 104, ALT, and AST should be defined

Data analysis

- In line 122: instead of “Time to treatment outcome…”, authors should say “time death…” given there are other outcomes apart from death
- The authors should make it clear that the outcome variable was binary, how the binary outcome variable was defined and which outcome categories went into the “died” and “did not die”
- Authors should make it clear how patients LTFU were treated: died vs did not die
- Authors should give an indication on whether they tested for collinearity among predictor variables
- Line 126: The statement “The selection of variables for the multivariable model was based on their established association with TB mortality in prior literature” negates the value of bivariate analysis (Table 1), which should guide which variables should go in the multivariable model. Use of a variable known to be predicator in literature should only be picked in addition to those found to be significant in Table 1.
- Lines 112-130: the statement “To assess the 129 robustness of our findings, a sensitivity analysis was performed by re-running the final 130 multivariable Cox model before and after the exclusion of participants” is not clear. Authors should explicitly explain how they performed their sensitivity analysis, i.e., what manipulation was made to the dataset. “….exclusion of participants” is verge.

**Results**

- To refresh the leader, find a way of including the denominator (316) in lines 138/139.
- Line 153: include “CI” in “…..(n = 60; 14.8%–23.8%)”
- Line 155: include p-value in “….bacteriological TB had a lower mortality rate compared to those diagnosed clinically (14.6% vs.156 26.4 %)”.
- Table 1: Age should be grouped to assess if there are differences by age category
- Table: In the methods section, including figure 1, the authors gave an indication that they performed a complete case analysis (though not explicitly stated). However, in Table 1, the value of “n” for some categories (e.g., HIV status, Diagnosis type, and DOT plan) does not add up to 316 (the total number of cases analyzed). These inconsistencies extend to the other columns. For example, the number of deaths by sex adds up to 57 and not the 60 stated in line 153. PLHIV has 58 deaths, Type of TB has 60; Type of patient has 59 and Diagnosis type has 58. Authors should correct these inconsistencies.
- Line 164-168 should state the denominator (total number of deaths)
- Line 167-168 says “…The majority of deaths occurred within the first 30 days (1 month)…” yet Table 2 shows that only 40.3% of deaths occurred in the first 30 days. It may be correct to say majority (51.6%) of deaths occurred in the first 60 days.
- Table 2 has a total of 61 deaths, yet line 153 says there were 60 deaths. Please correct this inconsistency.
- Line 190-202: It is not clear in this section or in the data analysis section how variables that were included in the multivariate analysis were selected. For example, how was HIV status, which was not significant at bivariate analysis, included in the multivariate analysis? The method of loading variables in the model may also need to be explicitly stated: at once, forward, or backward.
- The sensitivity analysis that the authors stated in the methods section (lines 128-130), does not appear in the results section

**Discussion and conclusion**

- No comment for now until the above comments are addressed.

**References**

- Some references are repeated, e.g. 5,7 & 11 relate to the same study, just as 8 &10
