# Supplementary material for: Predictors of tuberculosis mortality among patients on treatment: A retrospective cohort study at a rural general hospital in Zambia
Source: PLoS One. 2026 Jun 24;21(6):e0345056. doi: 10.1371/journal.pone.0345056 (PMC13293434; doi:10.1371/journal.pone.0345056)
Supplement: Attachment — Submitted filename: Response_to_Reviewers_auresp_2.docx [file pone.0345056.s006.docx]

Dr. Lukundo Siame

Luena Road No 21226, Batoka Area,

Livingstone Phone: 0976304745

Email: lukundosiame23@gmail.com Date: 23-09-2024

12 ^th^ February, 2025

To the Editor, PLOS ONE,

Dear Editor,

**Ref: Submission of a revised manuscript .**

We would like to thank the reviewers and PLos One editor and staff for taking the time to make suggestions that have improved our manuscript. We have now made all comments addressed by the reviewers. Below are point by point response to reviewer comments

Journal Requirements:

1. If the reviewer comments include a recommendation to cite specific previously published works, please review and evaluate these publications to determine whether they are relevant and should be cited. There is no requirement to cite these works unless the editor has indicated otherwise.

**Response**: not applicable

2. Please review your reference list to ensure that it is complete and correct. If you have cited papers that have been retracted, please include the rationale for doing so in the manuscript text, or remove these references and replace them with relevant current references. Any changes to the reference list should be mentioned in the rebuttal letter that accompanies your revised manuscript. If you need to cite a retracted article, indicate the article’s retracted status in the References list and also include a citation and full reference for the retraction notice.
**Response:** no references have been changed

Reviewer #1: The authors have made a great effort to address the issues from the previous review, and the manuscript has improved noticeably in clarity, transparency, and consistency between the objectives, methodology, and results. The survival analyses are appropriate, the outcome definition is clearer, and important restrictions pertaining to normal programmatic data are more openly addressed.

**Response:** thank you, for your kind words

However, there are still some issues. The lack of sensitivity or robustness studies reduces confidence in this finding, as the relationship between DOT modality and mortality is still susceptible to residual confounding

**Response:** Thank you for this important observation. We acknowledge that sensitivity analyses were not performed, primarily due to the limitations inherent in routinely collected programmatic data. However, to strengthen the robustness of our findings, we assessed model stability through evaluation of collinearity, proportional hazards assumptions, and purposeful variable selection based on prior evidence and clinical relevance. These steps were undertaken to improve the reliability of the final model estimates.

. Additionally, some estimates should be interpreted cautiously due to the very small number of occurrences relative to the number of variables. The discussion and conclusions would benefit from more careful wording to avoid implying causal relationships.

Overall, this is a technically sound and relevant study, and addressing these remaining points would further strengthen the manuscript

**Response**: Thank you for this comment. We have revised the discussion section to remove or rephrase sentences that could imply causal relationships. Specifically, we have modified statements to reflect associations rather than causation. Examples include:

“This study found that individuals with TB on a facility-based Directly Observed Treatment (DOT) plan were associated with a higher risk of mortality compared to those on home-based DOT.”
 “This study found that tuberculosis-related mortality was higher among individuals living with HIV compared to those who were HIV-negative.”
 “This study found that increasing age was associated with higher mortality.”

**Reviewer #2:** General comment
Author have addressed almost all the comments I raised. The article reads far much better. I however, have a few additional comments that authors may need to address before the article can be considered for publication.

Abstract
- May not need to include “without bacteriological confirmation” in brackets after “clinically diagnosed TB.”

**Response:** thank you for your suggestion , the word without bacteriological confirmation has been deleted.

Methods
Eligibility and Sampling Methods
- Please ensure that the sampling method used is explicitly stated. What sampling method did you use to select Maamba General Hospital? And what was the rationale? What sampling method did you use to select the actual participants?

- Study population needs to be explicitly stated. For this reason, I suggest calling the subsection ‘Study population and sampling methods. Inclusion and exclusion criteria can be a separate section study and sampling methods and a separate section	for inclusion criteria to be clearer and explicitly

**Response:** thank you for your suggestion. We have now created two separate section one

Data analysis

- Line one 182 “A purposeful selection strategy” should be replaced with ‘enter method.’

**Response:** thank you. we have replaced purpose selection strategy to enter methods
Results
Predictors of TB mortality
- I suggest leaving out the text in lines 259 – 264. What the reader is interested in are adjusted results. Details on crude ratios can be left to table 3

**Response:** thank you for your suggestion. We have deleted that section.

Discussion
- In line 286 – “…and some may have had alternative conditions that mimic TB”. Can we replace ‘alternative conditions’ with a more “scientific” terminology?

**Response:** thank you . we have change to a more scientific term “other differential diagnosis”

We would like to thank the reviewer for evaluating this work once more.

Dr. Lukundo Siame

On behalf of co authors

Livingstone university teaching Hospital
