# Supplementary material for: Predictors of tuberculosis mortality among patients on treatment: A retrospective cohort study at a rural general hospital in Zambia
Source: PLoS One. 2026 Jun 24;21(6):e0345056. doi: 10.1371/journal.pone.0345056 (PMC13293434; doi:10.1371/journal.pone.0345056)
Supplement: Peer review history

# Peer review: Decision Letter 0

**DOI:** 10.1371/journal.pone.0345056.r001

**Authors:** Graeme Hoddinott

6 Jan 2026

PONE-D-25-56555

Predictors of Tuberculosis Mortality Among Patients on Treatment: A Retrospective Cohort Study at a Rural General Hospital in Zambia

PLOS One

Dear Dr. Siame,

Thank you for submitting your manuscript to PLOS ONE. After careful consideration, we feel that it has merit but does not fully meet PLOS ONE’s publication criteria as it currently stands. Therefore, we invite you to submit a revised version of the manuscript that addresses the points raised during the review process.

==============================

**ACADEMIC EDITOR:**

The reviewers have raised a number of substantial issues for you to address. To my reading, I believe that these can be addressed with editing, and therefore invite you to do so.

==============================

Please submit your revised manuscript by Feb 20 2026 11:59PM. If you will need more time than this to complete your revisions, please reply to this message or contact the journal office at plosone@plos.org. When you're ready to submit your revision, log on to https://www.editorialmanager.com/pone/ and select the 'Submissions Needing Revision' folder to locate your manuscript file.

Please include the following items when submitting your revised manuscript:

- A letter that responds to each point raised by the academic editor and reviewer(s). You should upload this letter as a separate file labeled 'Response to Reviewers'.
- A marked-up copy of your manuscript that highlights changes made to the original version. You should upload this as a separate file labeled 'Revised Manuscript with Track Changes'.
- An unmarked version of your revised paper without tracked changes. You should upload this as a separate file labeled 'Manuscript'.

If you would like to make changes to your financial disclosure, please include your updated statement in your cover letter. Guidelines for resubmitting your figure files are available below the reviewer comments at the end of this letter.

If applicable, we recommend that you deposit your laboratory protocols in protocols.io to enhance the reproducibility of your results. Protocols.io assigns your protocol its own identifier (DOI) so that it can be cited independently in the future. For instructions see: https://journals.plos.org/plosone/s/submission-guidelines#loc-laboratory-protocols. Additionally, PLOS ONE offers an option for publishing peer-reviewed Lab Protocol articles, which describe protocols hosted on protocols.io. Read more information on sharing protocols at https://plos.org/protocols?utm_medium=editorial-email&utm_source=authorletters&utm_campaign=protocols.

We look forward to receiving your revised manuscript.

Kind regards,

Graeme Hoddinott, Ph.D

Academic Editor

PLOS One

Journal requirements:

When submitting your revision, we need you to address these additional requirements.

1. Please ensure that your manuscript meets PLOS ONE's style requirements, including those for file naming. The PLOS ONE style templates can be found at

https://journals.plos.org/plosone/s/file?id=wjVg/PLOSOne_formatting_sample_main_body.pdf and

https://journals.plos.org/plosone/s/file?id=ba62/PLOSOne_formatting_sample_title_authors_affiliations.pdf

2. We note that there is identifying data in the Supporting Information file < cleaned.xlsx>. Due to the inclusion of these potentially identifying data, we have removed this file from your file inventory. Prior to sharing human research participant data, authors should consult with an ethics committee to ensure data are shared in accordance with participant consent and all applicable local laws.

Data sharing should never compromise participant privacy. It is therefore not appropriate to publicly share personally identifiable data on human research participants. The following are examples of data that should not be shared:

-Name, initials, physical address

-Ages more specific than whole numbers

-Internet protocol (IP) address

-Specific dates (birth dates, death dates, examination dates, etc.)

-Contact information such as phone number or email address

-Location data

-ID numbers that seem specific (long numbers, include initials, titled “Hospital ID”) rather than random (small numbers in numerical order)

Data that are not directly identifying may also be inappropriate to share, as in combination they can become identifying. For example, data collected from a small group of participants, vulnerable populations, or private groups should not be shared if they involve indirect identifiers (such as sex, ethnicity, location, etc.) that may risk the identification of study participants.

Additional guidance on preparing raw data for publication can be found in our Data Policy (https://journals.plos.org/plosone/s/data-availability#loc-human-research-participant-data-and-other-sensitive-data) and in the following article: http://www.bmj.com/content/340/bmj.c181.long.

Please remove or anonymize all personal information, ensure that the data shared are in accordance with participant consent, and re-upload a fully anonymized data set. Please note that spreadsheet columns with personal information must be removed and not hidden as all hidden columns will appear in the published file.

If the reviewer comments include a recommendation to cite specific previously published works, please review and evaluate these publications to determine whether they are relevant and should be cited. There is no requirement to cite these works unless the editor has indicated otherwise.

[Note: HTML markup is below. Please do not edit.]

Reviewers' comments:

Reviewer's Responses to Questions

**Comments to the Author**

1. Is the manuscript technically sound, and do the data support the conclusions?

The manuscript must describe a technically sound piece of scientific research with data that supports the conclusions. Experiments must have been conducted rigorously, with appropriate controls, replication, and sample sizes. The conclusions must be drawn appropriately based on the data presented.

Reviewer #1: Yes

Reviewer #2: Yes

**********

2. Has the statistical analysis been performed appropriately and rigorously?

Reviewer #1: Yes

Reviewer #2: No

**********

3. Have the authors made all data underlying the findings in their manuscript fully available?

The PLOS Data policy requires authors to make all data underlying the findings described in their manuscript fully available without restriction, with rare exception (please refer to the Data Availability Statement in the manuscript PDF file). The data should be provided as part of the manuscript or its supporting information, or deposited to a public repository. For example, in addition to summary statistics, the data points behind means, medians and variance measures should be available. If there are restrictions on publicly sharing data—e.g. participant privacy or use of data from a third party—those must be specified.

Reviewer #1: Yes

Reviewer #2: Yes

**********

4. Is the manuscript presented in an intelligible fashion and written in standard English?

PLOS ONE does not copyedit accepted manuscripts, so the language in submitted articles must be clear, correct, and unambiguous. Any typographical or grammatical errors should be corrected at revision, so please note any specific errors here.

Reviewer #1: Yes

Reviewer #2: Yes

**********

5. Review Comments to the Author

Please use the space provided to explain your answers to the questions above. You may also include additional comments for the author, including concerns about dual publication, research ethics, or publication ethics. (Please upload your review as an attachment if it exceeds 20,000 characters)

Reviewer #1: A. Major concerns (must be addressed)

Contradiction in results vs conclusion (critical).

The Results and Table 3 show family-based DOT is protective (aHR 0.42), i.e. family DOT associated with lower mortality compared to facility DOT. Yet the Conclusion (page ~284) states: “Facility-based DOT and higher weights were associated with reduced mortality.” This is a direct contradiction and must be corrected. The narrative must match the numeric results (or the results recalculated if the table is wrong).

Missing data handling not transparent / needs numbers.

The manuscript excludes 84/400 records for missing birth date, outcome etc (good to show flowchart), but the amount of missingness for each analysis variable (HIV status, weight, labs, CD4, investigations) is not reported. Provide a table of missingness (n, %) per variable and state how missing predictor values were handled in uni- and multivariable models (complete-case vs multiple imputation). If complete-case was used, report how many participants contributed to the multivariable model and compare characteristics of included vs excluded.

Selection of covariates for multivariable model insufficiently justified.

The methods say selection was based on prior literature but do not specify which variables were entered a priori. Provide (a) full list of variables considered for the multivariable model; (b) justification (clinical + statistical p-value threshold if used); and (c) the exact model-building strategy (enter all, backwards elimination, change-in-estimate). Also provide the number of events per variable to ensure model is not overfit. With 60 deaths, rule-of-thumb EPV suggests limiting covariates.

Proportional hazards testing requires details.

The manuscript states a Schoenfeld global test p>0.05 but does not give the test statistic, the p-value, or whether any individual covariates violated PH. Provide: global Schoenfeld p-value, and per-variable Schoenfeld test (or show plots). If any variable violates PH, present time-varying coefficients or stratified models.

Potential confounding by severity for DOT assignment.

The observed association between DOT type and mortality is likely confounded by indication: sicker patients may be placed on facility DOT. The authors acknowledge this but did not adjust for baseline disease severity. Key missing severity proxies: hospitalization at treatment start (inpatient vs outpatient), chest X-ray findings, oxygen saturation, presence of disseminated TB, baseline weight/BMI (they have weight), performance status, and smear/GeneXpert bacillary load. If these variables exist, include them in the adjusted model.

Time scale & person-years calculation needs clarity.

Authors report incidence rate = 42.1 per 100 person-years. Provide the denominator: total person-time of follow-up (sum of follow-up time) and show calculations (for example; 60 deaths / X person-years). Also confirm how they handled varying follow-up times (censoring rules) and whether transfers-out were censored at last visit.

Outcome definition: all-cause vs TB-specific mortality unclear.

The manuscript uses “mortality” but it is not explicit whether deaths were all-cause or attributed to TB. Clarify how cause of death was assigned (verbal, clinician, death certificate), and if only in-hospital deaths included. PLOS ONE requires clear outcome definitions.

Timing of data collection vs study period; dates inconsistent.

Methods state sample was patients who started treatment May 2019–Mar 2024; data collection “during the second and third weeks of September 2024”. But elsewhere there are citations dated 2025 in References. Just confirm dates and ensure no typographical errors.

B. Recommended additional / sensitivity analyses (analyses to strengthen causal interpretation and robustness)

Report number of events used in the multivariable model and EPV.

If EPV < 10 per predictor, consider using fewer covariates or penalized regression.

Provide per-variable Schoenfeld plots or scaled Schoenfeld residual p-values and, if any violations exist, fit time-varying covariates or present stratified Cox model.

Multiple imputation for missing covariates (if missingness >5–10% for important variables). Compare results with complete-case analysis.

Sensitivity analysis excluding early deaths (deaths in first 7–14 days) to test effect of misdiagnosis / late presentation (authors mention many deaths in first month). If many early deaths are due to other diseases misdiagnosed as TB, associations may change.

Propensity-score adjustment or inverse probability weighting (IPW) to reduce confounding for DOT assignment (facility vs family). Use available covariates (age, weight, HIV, diagnosis type, inpatient status) to estimate probability of facility DOT and then adjust.

Effect modification / interaction checks: explore interactions of HIV status × weight and HIV × diagnosis type (clinical vs bacteriologic), present stratified aHRs if interactions significant.

Cause-specific vs all-cause mortality: If available, present TB-attributed deaths separately or at least comment on how many deaths had alternative causes recorded.

Include ART status and timing for PLHIV.

Include ART use at TB treatment initiation and timing of ART initiation if available (these strongly influence mortality).

Model diagnostics: proportional hazards, influential observations, multicollinearity (VIFs), goodness-of-fit metrics.

Report absolute risks and number-needed-to-treat-type statements where relevant (absolute difference in 6-month mortality between clinical vs bacteriologically diagnosed patients).

C. Reporting, tables, figures, and editorial fixes

Tables / Figures;

Table 1: Improve formatting. Report N (and %) for missing values per variable. For continuous variables, ensure correct medians/IQR and units (creatinine units μmol/L consistent). Spell out denominators (PLHIV, n=300 — conflicts with n=316; explain why 16 missing).

Table 2 (time-to-death); The column headings and the “censored” values are confusing. Explain how survival probability is calculated for each interval and ensure the “Deaths (%)” is deaths in that interval / total deaths or / persons at risk? The table currently shows “24 (40.3)” for 0–30 days; clarify numerator/denominator. Also the p<0.001 under Table 2 appears misplaced, kindly clarify what test that refers to.

Table 3: Add columns for N included in each model (n with complete data). Report exact aHR (not rounded inconsistently) and include model baseline hazards if relevant.

Kaplan–Meier plots:

Add numbers at risk at bottom, add confidence bands, and label axes clearly (time in days, survival probability). Provide log-rank p-values on the plots. Current Figures 3 and 4 mention p-values but axes tick marks and labels could be clearer.

Flow diagram:

Figure 1 should show the total records reviewed (400), exclusions (84; list reasons and counts), final n=316 but ensure the graphic is high-resolution and matches numbers in text.

Variable definitions:

Define clinically diagnosed TB precisely (criteria used: clinical + X-ray? urine LAM only? empirical treatment?). Define DOT categories explicitly (what qualifies as family-based DOT?). Define “treatment outcomes” per national guidelines.

Laboratory timing:

State when labs were measured (baseline tests within how many days of treatment start?). For CD4 counts give numerator because earlier Table 1 indicates CD4 median but PLHIV n=100.

Ethics & Consent:

Ethics approval details are provided; add explicit statement on waiver of consent for retrospective record review if that was the case.

Data availability:

Ensure the S2 cleaned.xlsx is attached and contains all variables needed to reproduce analyses; mention repository or include DOI.

References / duplication:

There are duplicated references (Bwembya et al. appears multiple times with slight differences). Clean and ensure correct formatting per PLOS ONE style. Also watch for some citations dated 2025/2024 — ensure accuracy.

Grammar / phrasing:

Fix a number of typos and grammatical slips (double periods “..”, sentence grammar in Results and Conclusion). Reword overly causal language (replace “influenced” with “associated”).

D. Specific minor points and suggested text edits

Correct the Conclusion sentence;

Current problematic sentence: “Facility-based DOT and higher weights were associated with reduced mortality.”

Suggested: “Family-based DOT and higher baseline weight were associated with reduced mortality; facility-based DOT was associated with higher mortality in crude analyses, though this may reflect confounding by indication.” (Only use if supported by adjusted model.)

Provide exact Schoenfeld test outputs in Methods or Results,

“Schoenfeld global test: χ² = X.X, p = 0.XX; per-variable tests: age p = 0.XX, DOT p = 0.XX. No significant violations were observed.”

Person-time calculation example to include;

“Total person-time of follow-up was X.X person-years (316 participants, median follow-up 174 days); incidence rate = 60 deaths / X.X py = 42.1 per 100 py.” (Authors must fill X.X).

Clarify n used in lab summaries: CD4 is only for PLHIV — state that median CD4 (n = 100) = 331 cells/mm³.

Reviewer #2: The paper address a very important topic in TB programing. It also adopts and appropriate methodology. However, a number of issues need to be addressed before the paper can be published. A section-by-section feedback is provided in the attached file.

**********

6. PLOS authors have the option to publish the peer review history of their article (what does this mean?). If published, this will include your full peer review and any attached files.

If you choose “no”, your identity will remain anonymous but your review may still be made public.

**Do you want your identity to be public for this peer review?**  For information about this choice, including consent withdrawal, please see our Privacy Policy.

Reviewer #1: **Yes:** Marshall C Mubanga

Reviewer #2: **Yes:** Josphat Bwembya

**********

[NOTE: If reviewer comments were submitted as an attachment file, they will be attached to this email and accessible via the submission site. Please log into your account, locate the manuscript record, and check for the action link "View Attachments". If this link does not appear, there are no attachment files.]

To ensure your figures meet our technical requirements, please review our figure guidelines: https://journals.plos.org/plosone/s/figures

You may also use PLOS’s free figure tool, NAAS, to help you prepare publication quality figures: https://journals.plos.org/plosone/s/figures#loc-tools-for-figure-preparation.

NAAS will assess whether your figures meet our technical requirements by comparing each figure against our figure specifications.

Attachment Submitted filename: Reviewer Comments_Josphat Bwembya.docx

# Author comment: Author response to Decision Letter 1

**DOI:** 10.1371/journal.pone.0345056.r002

28 Jan 2026

Reviewer #1: A. Major concerns (must be addressed)

Contradiction in results vs conclusion (critical).

1. The Results and Table 3 show family-based DOT is protective (aHR 0.42), i.e. family DOT associated with lower mortality compared to facility DOT. Yet the Conclusion (page ~284) states: “Facility-based DOT and higher weights were associated with reduced mortality.” This is a direct contradiction and must be corrected. The narrative must match the numeric results (or the results recalculated if the table is wrong).

Response: thank you , for the observation , we have revised the conclusion to reflect the results provide

2. Missing data handling not transparent / needs numbers.

The manuscript excludes 84/400 records for missing birth date, outcome etc (good to show flowchart), but the amount of missingness for each analysis variable (HIV status, weight, labs, CD4, investigations) is not reported. Provide a table of missingness (n, %) per variable and state how missing predictor values were handled in uni- and multivariable models (complete-case vs multiple imputation). If complete-case was used, report how many participants contributed to the multivariable model and compare characteristics of included vs excluded.

Selection of covariates for multivariable model insufficiently justified.

Response: We thank the reviewer for this comment. We have now added a table summarising missing data for all study variables (Supplementary Table S1) and explicitly described how missing data were handled. Complete-case analysis was used due to low missingness among model covariates (<5%), and the final multivariable model included 284 participants. We have also clarified our rationale for covariate selection based on prior literature and clinical relevance.

3. The methods say selection was based on prior literature but do not specify which variables were entered a priori. Provide (a) full list of variables considered for the multivariable model; (b) justification (clinical + statistical p-value threshold if used); and (c) the exact model-building strategy (enter all, backwards elimination, change-in-estimate). Also provide the number of events per variable to ensure model is not overfit. With 60 deaths, rule-of-thumb EPV suggests limiting covariates.

Response: We have revised the Methods section to clarify covariate selection and model-building. Variables considered for multivariable analysis were age, sex, employment status, HIV status, tuberculosis type, diagnosis type, DOT plan, and weight at treatment initiation. Laboratory variables were considered but excluded due to substantial missingness.Variables were selected based on prior literature and clinical relevance, with additional consideration of variables associated with mortality at p < 0.20 in univariable analysis. Age and sex were retained a priori due to their known confounding effects. A purposeful selection strategy was used, with all selected variables entered simultaneously into the multivariable Cox regression model and no automated stepwise procedures applied. The final multivariable model included 284 participants with 54 deaths and 8 covariates, corresponding to approximately 7 events per variable, which is acceptable for observational survival analyses when variables are selected a priori. Sensitivity analyses were conducted to assess model robustness.

4. Proportional hazards testing requires details.

The manuscript states a Schoenfeld global test p>0.05 but does not give the test statistic, the p-value, or whether any individual covariates violated PH. Provide: global Schoenfeld p-value, and per-variable Schoenfeld test (or show plots). If any variable violates PH, present time-varying coefficients or stratified models.

Response: Thank you for this important methodological comment. We have revised the Methods section to provide full details of the proportional hazards assumption testing.The proportional hazards assumption was assessed using Schoenfeld residuals after fitting the multivariable Cox model. The global Schoenfeld test showed no evidence of violation of the proportional hazards assumption (χ² = 4.58, df = 8, p = 0.801). In addition, all individual covariates satisfied the proportional hazards assumption, with per-variable Schoenfeld tests yielding p-values > 0.05. These results are now explicitly reported in the Methods section, and the per-covariate Schoenfeld test results have been added as Supplementary Table S2. As no violations were detected, time-varying coefficients or stratified Cox models were not required.

5. Potential confounding by severity for DOT assignment.

The observed association between DOT type and mortality is likely confounded by indication: sicker patients may be placed on facility DOT. The authors acknowledge this but did not adjust for baseline disease severity. Key missing severity proxies: hospitalization at treatment start (inpatient vs outpatient), chest X-ray findings, oxygen saturation, presence of disseminated TB, baseline weight/BMI (they have weight), performance status, and smear/GeneXpert bacillary load. If these variables exist, include them in the adjusted model.

Time scale & person-years calculation needs clarity.

Response: We agree with the reviewer that confounding by indication is a potential concern in the observed association between DOT type and mortality. Several recommended severity proxies were not available in the routine TB treatment registers used for this retrospective study, including hospitalization status at treatment initiation, chest radiographic findings, oxygen saturation, functional/performance status, and quantitative bacillary load. As such, these variables could not be included in the adjusted model. However, we did adjust for baseline weight, disseminated TB, HIV status, age, and diagnostic method (clinical vs bacteriological), which partially capture disease severity at presentation. Importantly, baseline weight remained independently protective after adjustment, supporting its role as a severity proxy.

To transparently address this limitation, we have:

1. Explicitly acknowledged confounding by indication in both the Discussion and Limitations sections.

2. Clarified that the association between facility-based DOT and higher mortality should not be interpreted causally.

3. Emphasized that patients assigned to facility-based DOT are likely sicker or lack adequate social support, which may explain the observed association.

We now explicitly state that future studies incorporating detailed clinical severity indicators are needed to more definitively assess the causal impact of DOT modality on mortality.

6. Authors report incidence rate = 42.1 per 100 person-years. Provide the denominator: total person-time of follow-up (sum of follow-up time) and show calculations (for example; 60 deaths / X person-years). Also confirm how they handled varying follow-up times (censoring rules) and whether transfers-out were censored at last visit.

Response: Thank you for this comment. All participants were followed from treatment initiation until death or censoring at treatment completion, loss to follow-up, or administrative censoring at 12 months. The cohort contributed 51,968 person-days (142.3 person-years) of follow-up, during which 60 deaths occurred, yielding an incidence rate of 42.1 deaths per 100 person-years. These details have now been clarified in the Methods and Results sections.

7. Outcome definition: all-cause vs TB-specific mortality unclear.

The manuscript uses “mortality” but it is not explicit whether deaths were all-cause or attributed to TB. Clarify how cause of death was assigned (verbal, clinician, death certificate), and if only in-hospital deaths included. PLOS ONE requires clear outcome definitions.

Response : Thank you for this important clarification. The outcome in this study was all-cause mortality during tuberculosis treatment, not TB-specific mortality. Deaths were identified from the routine TB treatment register and recorded by clinicians as part of standard programmatic follow-up. Formal cause-of-death determination (e.g., death certificates or verbal autopsy) was not available. Both in-hospital and out-of-hospital deaths reported during follow-up were included. We have now explicitly clarified the outcome definition in the Methods section.

8. Timing of data collection vs study period; dates inconsistent.

Methods state sample was patients who started treatment May 2019–Mar 2024; data collection “during the second and third weeks of September 2024”. But elsewhere there are citations dated 2025 in References. Just confirm dates and ensure no typographical errors.

Response: Thank you for raising this point. We confirm that the study included patients who initiated tuberculosis treatment between May 2019 and March 2024. Data collection was conducted retrospectively during the second and third weeks of September 2024 through abstraction of routine TB treatment registers. References published in 2025 were cited to contextualize the findings and do not reflect data collection beyond the study period. We have clarified the timing of data collection in the Methods section to avoid any confusion.

9. B. Recommended additional / sensitivity analyses (analyses to strengthen causal interpretation and robustness)

Report number of events used in the multivariable model and EPV.

If EPV < 10 per predictor, consider using fewer covariates or penalized regression.

Response: We have now reported the number of events and EPV in the multivariable model. The adjusted analysis included 60 deaths and 8 prespecified covariates (EPV ≈ 7.5). Although below the traditional EPV threshold of 10, predictors were selected a priori based on clinical relevance, and model estimates were stable. This has been clarified in the Methods and Limitations sections.

10. Provide per-variable Schoenfeld plots or scaled Schoenfeld residual p-values and, if any violations exist, fit time-varying covariates or present stratified Cox model.

Multiple imputation for missing covariates (if missingness >5–10% for important variables). Compare results with complete-case analysis.

Response: thank you for the comment. We assessed the proportional hazards assumption using Schoenfeld residuals. The global test showed no evidence of violation (χ² = 4.58, p = 0.801), and all individual covariates satisfied the proportional hazards assumption; per-variable Schoenfeld test results are provided in the Supplementary material. Missingness among covariates included in the multivariable model was low (<6%), with no missing outcome data; therefore, complete-case analysis was used and multiple imputation was not performed.

11. Sensitivity analysis excluding early deaths (deaths in first 7–14 days) to test effect of misdiagnosis / late presentation (authors mention many deaths in first month). If many early deaths are due to other diseases misdiagnosed as TB, associations may change.

Response: Thank you for this thoughtful suggestion. We agree that deaths occurring very early after treatment initiation may reflect late presentation or possible misdiagnosis. We considered a sensitivity analysis excluding deaths in the first 7–14 days; however, given the limited number of events and the modest events-per-variable ratio, excluding early deaths would substantially reduce statistical power and risk model instability. We have therefore addressed this issue by explicitly discussing the potential influence of early deaths and misdiagnosis in the Discussion section and by interpreting our findings with appropriate caution. Future studies with larger sample sizes and more detailed diagnostic data would be better suited to formally evaluate this sensitivity analysis.

12. Propensity-score adjustment or inverse probability weighting (IPW) to reduce confounding for DOT assignment (facility vs family). Use available covariates (age, weight, HIV, diagnosis type, inpatient status) to estimate probability of facility DOT and then adjust.

Response: We acknowledge the suggestion to use propensity score methods or inverse probability weighting to address confounding in DOT assignment. However, we did not apply these methods because our primary analysis used multivariable Cox regression with covariates selected a priori based on clinical relevance. Additionally, key indicators of baseline disease severity influencing DOT assignment were not fully available, limiting the ability of propensity score methods to adequately address confounding by indication. We therefore used a transparent regression-based approach and acknowledge the possibility of residual confounding in the limitations.

13. Effect modification / interaction checks: explore interactions of HIV status × weight and HIV × diagnosis type (clinical vs bacteriologic), present stratified aHRs if interactions significant.

Response: We acknowledge the reviewer’s suggestion regarding assessment of effect modification using interaction terms. However, given the limited number of mortality events in the cohort, the study was not adequately powered to reliably assess interaction effects or to present stratified adjusted hazard ratios. We therefore did not pursue formal interaction analyses and have focused on reporting main effects from the multivariable Cox regression models. This has been noted as a limitation of the study.

14. Cause-specific vs all-cause mortality: If available, present TB-attributed deaths separately or at least comment on how many deaths had alternative causes recorded.

Response: thank you, With respect to mortality classification, the TB treatment registers recorded all-cause mortality only, and cause-specific attribution of death was not consistently documented. Consequently, cause-specific mortality analyses were not feasible. This limitation has been acknowledged in the Methods and Discussion sections.

15. Include ART status and timing for PLHIV.

Include ART use at TB treatment initiation and timing of ART initiation if available (these strongly influence mortality).

Response: We agree that antiretroviral therapy (ART) status and the timing of ART initiation are important determinants of mortality among people living with HIV. However, in the Zambian TB program, the timing of ART initiation is not routinely recorded in TB treatment registers or standard clinical records. Consequently, ART use at TB treatment initiation and ART timing were not available for this retrospective analysis and could not be included. This limitation has been clearly acknowledged in the Discussion.

16. Model diagnostics: proportional hazards, influential observations, multicollinearity (VIFs), goodness-of-fit metrics.

Report absolute risks and number-needed-to-treat-type statements where relevant (absolute difference in 6-month mortality between clinical vs bacteriologically diagnosed patients).

Response : We thank the reviewer for this suggestion. The proportional hazards assumption was assessed using Schoenfeld residuals and no violations were observed. Given that the model was specified a priori with a limited number of clinically relevant covariates and showed no evidence of instability, additional diagnostics such as assessment of influential observations and multicollinearity were not performed. Formal goodness-of-fit measures for Cox models are limited; therefore, model adequacy was primarily evaluated through assumption checks. With respect to absolute risks, the study focused on identifying predictors of mortality using time-to-event methods. While crude differences in mortality between clinical and bacteriologically diagnosed patients are reported, number-needed-to-treat–type measures were not calculated, as these may be misleading in non-randomized survival analyses.

C. Reporting, tables, figures, and editorial fixes

Tables / Figures;

17. Table 1: Improve formatting. Report N (and %) for missing values per variable. For continuous variables, ensure correct medians/IQR and units (creatinine units μmol/L consistent). Spell out denominators (PLHIV, n=300 — conflicts with n=316; explain why 16 missing).

Response: Table 1 was revised to improve clarity and consistency. We now report the number and percentage of missing values for each variable, prese

Attachment Submitted filename: Response to Reviewers.docx

# Peer review: Decision Letter 1

**DOI:** 10.1371/journal.pone.0345056.r003

**Authors:** Graeme Hoddinott

10 Feb 2026

PONE-D-25-56555R1

Predictors of Tuberculosis Mortality Among Patients on Treatment: A Retrospective Cohort Study at a Rural General Hospital in Zambia

PLOS One

Dear Dr. Siame,

Thank you for submitting your manuscript to PLOS ONE. After careful consideration, we feel that it has merit but does not fully meet PLOS ONE’s publication criteria as it currently stands. Therefore, we invite you to submit a revised version of the manuscript that addresses the points raised during the review process.

==============================

**ACADEMIC EDITOR:**

The reviewers have raised a few further suggestions on how to represent your work optimally.

==============================

Please submit your revised manuscript by Mar 27 2026 11:59PM. If you will need more time than this to complete your revisions, please reply to this message or contact the journal office at plosone@plos.org. When you're ready to submit your revision, log on to https://www.editorialmanager.com/pone/ and select the 'Submissions Needing Revision' folder to locate your manuscript file.

Please include the following items when submitting your revised manuscript:

- A letter that responds to each point raised by the academic editor and reviewer(s). You should upload this letter as a separate file labeled 'Response to Reviewers'.
- A marked-up copy of your manuscript that highlights changes made to the original version. You should upload this as a separate file labeled 'Revised Manuscript with Track Changes'.
- An unmarked version of your revised paper without tracked changes. You should upload this as a separate file labeled 'Manuscript'.

-->If you would like to make changes to your financial disclosure, please include your updated statement in your cover letter. Guidelines for resubmitting your figure files are available below the reviewer comments at the end of this letter.

If applicable, we recommend that you deposit your laboratory protocols in protocols.io to enhance the reproducibility of your results. Protocols.io assigns your protocol its own identifier (DOI) so that it can be cited independently in the future. For instructions see: https://journals.plos.org/plosone/s/submission-guidelines#loc-laboratory-protocols. Additionally, PLOS ONE offers an option for publishing peer-reviewed Lab Protocol articles, which describe protocols hosted on protocols.io. Read more information on sharing protocols at https://plos.org/protocols?utm_medium=editorial-email&utm_source=authorletters&utm_campaign=protocols.

We look forward to receiving your revised manuscript.

Kind regards,

Graeme Hoddinott, Ph.D

Academic Editor

PLOS One

Journal Requirements:

1. If the reviewer comments include a recommendation to cite specific previously published works, please review and evaluate these publications to determine whether they are relevant and should be cited. There is no requirement to cite these works unless the editor has indicated otherwise.

2. Please review your reference list to ensure that it is complete and correct. If you have cited papers that have been retracted, please include the rationale for doing so in the manuscript text, or remove these references and replace them with relevant current references. Any changes to the reference list should be mentioned in the rebuttal letter that accompanies your revised manuscript. If you need to cite a retracted article, indicate the article’s retracted status in the References list and also include a citation and full reference for the retraction notice.

[Note: HTML markup is below. Please do not edit.]

Reviewers' comments:

Reviewer's Responses to Questions

**Comments to the Author**

1. If the authors have adequately addressed your comments raised in a previous round of review and you feel that this manuscript is now acceptable for publication, you may indicate that here to bypass the “Comments to the Author” section, enter your conflict of interest statement in the “Confidential to Editor” section, and submit your "Accept" recommendation.

Reviewer #1: All comments have been addressed

Reviewer #2: (No Response)

**********

2. Is the manuscript technically sound, and do the data support the conclusions?

The manuscript must describe a technically sound piece of scientific research with data that supports the conclusions. Experiments must have been conducted rigorously, with appropriate controls, replication, and sample sizes. The conclusions must be drawn appropriately based on the data presented.

Reviewer #1: Yes

Reviewer #2: Yes

**********

3. Has the statistical analysis been performed appropriately and rigorously?

Reviewer #1: Yes

Reviewer #2: Yes

**********

4. Have the authors made all data underlying the findings in their manuscript fully available?

The PLOS Data policy requires authors to make all data underlying the findings described in their manuscript fully available without restriction, with rare exception (please refer to the Data Availability Statement in the manuscript PDF file). The data should be provided as part of the manuscript or its supporting information, or deposited to a public repository. For example, in addition to summary statistics, the data points behind means, medians and variance measures should be available. If there are restrictions on publicly sharing data—e.g. participant privacy or use of data from a third party—those must be specified.

Reviewer #1: Yes

Reviewer #2: Yes

**********

5. Is the manuscript presented in an intelligible fashion and written in standard English?

PLOS ONE does not copyedit accepted manuscripts, so the language in submitted articles must be clear, correct, and unambiguous. Any typographical or grammatical errors should be corrected at revision, so please note any specific errors here.

Reviewer #1: Yes

Reviewer #2: Yes

**********

6. Review Comments to the Author

Please use the space provided to explain your answers to the questions above. You may also include additional comments for the author, including concerns about dual publication, research ethics, or publication ethics. (Please upload your review as an attachment if it exceeds 20,000 characters)

Reviewer #1: The authors have made a great effort to address the issues from the previous review, and the manuscript has improved noticeably in clarity, transparency, and consistency between the objectives, methodology, and results. The survival analyses are appropriate, the outcome definition is more clear, and important restrictions pertaining to normal programmatic data are more openly addressed.

However, there are still some issues. The lack of sensitivity or robustness studies reduces confidence in this finding, as the relationship between DOT modality and mortality is still susceptible to residual confounding. Additionally, some estimates should be interpreted cautiously due to the very small number of occurrences relative to the number of variables. The discussion and conclusions would benefit from more careful wording to avoid implying causal relationships.

Overall, this is a technically sound and relevant study, and addressing these remaining points would further strengthen the manuscript

Reviewer #2: General comment

Author have addressed almost all the comments I raised. The article reads far much better. I however, have a few additional comments that authors may need to address before the article can be considered for publication.

Abstract

- May not need to include “without bacteriological confirmation” in brackets after “clinically diagnosed TB.”

Methods

Eligibility and Sampling Methods

- Please ensure that the sampling method used is explicitly stated. What sampling method did you use to select Maamba General Hospital? And what was the rationale? What sampling method did you use to select the actual participants?

- Study population needs to be explicitly stated. For this reason, I suggest calling the subsection ‘Study population and sampling methods’. Inclusion and exclusion criteria can be a separate section

Data analysis

- Line one 182 “A purposeful selection strategy” should be replaced with ‘enter method.’

Results

Predictors of TB mortality

- I suggest leaving out the text in lines 259 – 264. What the reader is interested in are adjusted results. Details on crude ratios can be left to table 3

Discussion

- In line 286 – “…and some may have had alternative conditions that mimic TB”. Can we replace ‘alternative conditions’ with a more “scientific” terminology?

**********

7. PLOS authors have the option to publish the peer review history of their article (what does this mean?). If published, this will include your full peer review and any attached files.

If you choose “no”, your identity will remain anonymous but your review may still be made public.

**Do you want your identity to be public for this peer review?**  For information about this choice, including consent withdrawal, please see our Privacy Policy.

Reviewer #1: **Yes:** Marshall C Mubanga

Reviewer #2: **Yes:** Josphat Bwembya

**********

[NOTE: If reviewer comments were submitted as an attachment file, they will be attached to this email and accessible via the submission site. Please log into your account, locate the manuscript record, and check for the action link "View Attachments". If this link does not appear, there are no attachment files.]

To ensure your figures meet our technical requirements, please review our figure guidelines: https://journals.plos.org/plosone/s/figures

You may also use PLOS’s free figure tool, NAAS, to help you prepare publication quality figures: https://journals.plos.org/plosone/s/figures#loc-tools-for-figure-preparation.

NAAS will assess whether your figures meet our technical requirements by comparing each figure against our figure specifications.

# Author comment: Author response to Decision Letter 2

**DOI:** 10.1371/journal.pone.0345056.r004

12 Feb 2026

Dr. Lukundo Siame

Luena Road No 21226, Batoka Area,

Livingstone Phone: 0976304745

Email: lukundosiame23@gmail.com Date: 23-09-2024

12 th February, 2025

To the Editor, PLOS ONE,

Dear Editor,

Ref: Submission of a revised manuscript .

We would like to thank the reviewers and PLos One editor and staff for taking the time to make suggestions that have improved our manuscript. We have now made all comments addressed by the reviewers. Below are point by point response to reviewer comments

Journal Requirements:

1. If the reviewer comments include a recommendation to cite specific previously published works, please review and evaluate these publications to determine whether they are relevant and should be cited. There is no requirement to cite these works unless the editor has indicated otherwise.

Response: not applicable

2. Please review your reference list to ensure that it is complete and correct. If you have cited papers that have been retracted, please include the rationale for doing so in the manuscript text, or remove these references and replace them with relevant current references. Any changes to the reference list should be mentioned in the rebuttal letter that accompanies your revised manuscript. If you need to cite a retracted article, indicate the article’s retracted status in the References list and also include a citation and full reference for the retraction notice.

Response: no references have been changed

Reviewer #1: The authors have made a great effort to address the issues from the previous review, and the manuscript has improved noticeably in clarity, transparency, and consistency between the objectives, methodology, and results. The survival analyses are appropriate, the outcome definition is clearer, and important restrictions pertaining to normal programmatic data are more openly addressed.

Response: thank you, for your kind words

However, there are still some issues. The lack of sensitivity or robustness studies reduces confidence in this finding, as the relationship between DOT modality and mortality is still susceptible to residual confounding

Response: Thank you for this important observation. We acknowledge that sensitivity analyses were not performed, primarily due to the limitations inherent in routinely collected programmatic data. However, to strengthen the robustness of our findings, we assessed model stability through evaluation of collinearity, proportional hazards assumptions, and purposeful variable selection based on prior evidence and clinical relevance. These steps were undertaken to improve the reliability of the final model estimates.

Additionally, some estimates should be interpreted cautiously due to the very small number of occurrences relative to the number of variables. The discussion and conclusions would benefit from more careful wording to avoid implying causal relationships.

Overall, this is a technically sound and relevant study, and addressing these remaining points would further strengthen the manuscript

Response: Thank you for this comment. We have revised the discussion section to remove or rephrase sentences that could imply causal relationships. Specifically, we have modified statements to reflect associations rather than causation. Examples include:

“This study found that individuals with TB on a facility-based Directly Observed Treatment (DOT) plan were associated with a higher risk of mortality compared to those on home-based DOT.”

“This study found that tuberculosis-related mortality was higher among individuals living with HIV compared to those who were HIV-negative.”

“This study found that increasing age was associated with higher mortality.”

Reviewer #2: General comment

Author have addressed almost all the comments I raised. The article reads far much better. I however, have a few additional comments that authors may need to address before the article can be considered for publication.

Abstract

- May not need to include “without bacteriological confirmation” in brackets after “clinically diagnosed TB.”

Response: thank you for your suggestion , the word without bacteriological confirmation has been deleted.

Methods

Eligibility and Sampling Methods

- Please ensure that the sampling method used is explicitly stated. What sampling method did you use to select Maamba General Hospital? And what was the rationale? What sampling method did you use to select the actual participants?

- Study population needs to be explicitly stated. For this reason, I suggest calling the subsection ‘Study population and sampling methods. Inclusion and exclusion criteria can be a separate section study and sampling methods and a separate section for inclusion criteria to be clearer and explicitly

Response: thank you for your suggestion. We have now created two separate section one

Data analysis

- Line one 182 “A purposeful selection strategy” should be replaced with ‘enter method.’

Response: thank you. we have replaced purpose selection strategy to enter methods

Results

Predictors of TB mortality

- I suggest leaving out the text in lines 259 – 264. What the reader is interested in are adjusted results. Details on crude ratios can be left to table 3

Response: thank you for your suggestion. We have deleted that section.

Discussion

- In line 286 – “…and some may have had alternative conditions that mimic TB”. Can we replace ‘alternative conditions’ with a more “scientific” terminology?

Response: thank you . we have change to a more scientific term “other differential diagnosis”

We would like to thank the reviewer for evaluating this work once more.

Dr. Lukundo Siame

On behalf of co authors

Livingstone university teaching Hospital

Attachment Submitted filename: Response_to_Reviewers_auresp_2.docx

# Peer review: Decision Letter 2

**DOI:** 10.1371/journal.pone.0345056.r005

**Authors:** Graeme Hoddinott

2 Mar 2026

Predictors of Tuberculosis Mortality Among Patients on Treatment: A Retrospective Cohort Study at a Rural General Hospital in Zambia

PONE-D-25-56555R2

Dear Dr. Siame,

We’re pleased to inform you that your manuscript has been judged scientifically suitable for publication and will be formally accepted for publication once it meets all outstanding technical requirements.

Within one week, you’ll receive an e-mail detailing the required amendments. When these have been addressed, you’ll receive a formal acceptance letter and your manuscript will be scheduled for publication.

An invoice will be generated when your article is formally accepted. Please note, if your institution has a publishing partnership with PLOS and your article meets the relevant criteria, all or part of your publication costs will be covered. Please make sure your user information is up-to-date by logging into Editorial Manager at Editorial Manager® and clicking the ‘Update My Information' link at the top of the page. For questions related to billing, please contact billing support.

If your institution or institutions have a press office, please notify them about your upcoming paper to help maximize its impact. If they’ll be preparing press materials, please inform our press team as soon as possible -- no later than 48 hours after receiving the formal acceptance. Your manuscript will remain under strict press embargo until 2 pm Eastern Time on the date of publication. For more information, please contact onepress@plos.org.

Kind regards,

Graeme Hoddinott, Ph.D

Academic Editor

PLOS One

Additional Editor Comments (optional):

Reviewers' comments:

Reviewer's Responses to Questions

**Comments to the Author**

1. If the authors have adequately addressed your comments raised in a previous round of review and you feel that this manuscript is now acceptable for publication, you may indicate that here to bypass the “Comments to the Author” section, enter your conflict of interest statement in the “Confidential to Editor” section, and submit your "Accept" recommendation.

Reviewer #1: All comments have been addressed

Reviewer #2: All comments have been addressed

**********

2. Is the manuscript technically sound, and do the data support the conclusions?

The manuscript must describe a technically sound piece of scientific research with data that supports the conclusions. Experiments must have been conducted rigorously, with appropriate controls, replication, and sample sizes. The conclusions must be drawn appropriately based on the data presented.

Reviewer #1: Yes

Reviewer #2: Yes

**********

3. Has the statistical analysis been performed appropriately and rigorously?

Reviewer #1: Yes

Reviewer #2: Yes

**********

4. Have the authors made all data underlying the findings in their manuscript fully available?

The PLOS Data policy requires authors to make all data underlying the findings described in their manuscript fully available without restriction, with rare exception (please refer to the Data Availability Statement in the manuscript PDF file). The data should be provided as part of the manuscript or its supporting information, or deposited to a public repository. For example, in addition to summary statistics, the data points behind means, medians and variance measures should be available. If there are restrictions on publicly sharing data—e.g. participant privacy or use of data from a third party—those must be specified.

Reviewer #1: Yes

Reviewer #2: Yes

**********

5. Is the manuscript presented in an intelligible fashion and written in standard English?

PLOS ONE does not copyedit accepted manuscripts, so the language in submitted articles must be clear, correct, and unambiguous. Any typographical or grammatical errors should be corrected at revision, so please note any specific errors here.

Reviewer #1: Yes

Reviewer #2: Yes

**********

6. Review Comments to the Author

Please use the space provided to explain your answers to the questions above. You may also include additional comments for the author, including concerns about dual publication, research ethics, or publication ethics. (Please upload your review as an attachment if it exceeds 20,000 characters)

Reviewer #1: (No Response)

Reviewer #2: The authors have addressed all my comments. I have no further comments. I take this opportunity congratute the authors on this important piece of work.

**********

7. PLOS authors have the option to publish the peer review history of their article (what does this mean?). If published, this will include your full peer review and any attached files.

If you choose “no”, your identity will remain anonymous but your review may still be made public.

**Do you want your identity to be public for this peer review?**  For information about this choice, including consent withdrawal, please see our Privacy Policy.

Reviewer #1: **Yes:** Marshall C Mubanga

Reviewer #2: **Yes:** Josphat Bwembya

**********

# Editor report: Acceptance letter

**DOI:** 10.1371/journal.pone.0345056.r006

**Authors:** Graeme Hoddinott

PONE-D-25-56555R2

PLOS One

Dear Dr. Siame,

I'm pleased to inform you that your manuscript has been deemed suitable for publication in PLOS One. Congratulations! Your manuscript is now being handed over to our production team.

At this stage, our production department will prepare your paper for publication. This includes ensuring the following:

* All references, tables, and figures are properly cited

* All relevant supporting information is included in the manuscript submission,

* There are no issues that prevent the paper from being properly typeset

You will receive further instructions from the production team, including instructions on how to review your proof when it is ready. Please keep in mind that we are working through a large volume of accepted articles, so please give us a few days to review your paper and let you know the next and final steps.

Lastly, if your institution or institutions have a press office, please let them know about your upcoming paper now to help maximize its impact. If they'll be preparing press materials, please inform our press team within the next 48 hours. Your manuscript will remain under strict press embargo until 2 pm Eastern Time on the date of publication. For more information, please contact onepress@plos.org.

You will receive an invoice from PLOS for your publication fee after your manuscript has reached the completed accept phase. If you receive an email requesting payment before acceptance or for any other service, this may be a phishing scheme. Learn how to identify phishing emails and protect your accounts at https://explore.plos.org/phishing.

If we can help with anything else, please email us at customercare@plos.org.

Thank you for submitting your work to PLOS ONE and supporting open access.

Kind regards,

PLOS ONE Editorial Office Staff

on behalf of

Dr. Graeme Hoddinott

Academic Editor

PLOS One
